# Analyzation of the Peripheral Blood Mononuclear Cells Atlas and Cell Communication of Rheumatoid Arthritis Patients Based on Single-Cell RNA-Seq

**DOI:** 10.1155/2023/6300633

**Published:** 2023-08-12

**Authors:** Xinqiang Song, Yu Zhang, Lijun Zhao, Jinke Fan, Tao Peng, Ying Ma, Nailiang Guo, Xiaotong Wang, Xudong Liu, Zhe Liu, Lei Wang

**Affiliations:** ^1^College of Life Sciences, Xinyang Normal University, Xinyang 464000, China; ^2^College of Medicine, Xinyang Normal University, Xinyang 464000, China; ^3^Xinyang Central Hospital, Xinyang 464000, China; ^4^School of Medicine, Chongqing University, Chongqing 400044, China; ^5^Chongqing Key Laboratory of Translational Research for Cancer Metastasis and Individualized Treatment, Chongqing University Cancer Hospital, Chongqing 400030, China; ^6^Department of Computer Science, City University of Hong Kong, Hong Kong, China

## Abstract

**Background:**

Rheumatoid arthritis (RA) is a common chronic inflammatory autoimmune disease with a multifactorial etiology. Peripheral blood is the main channel of the immune system, and peripheral blood mononuclear cells (PBMCs) are the immune cells that initiate the autoimmune inflammatory process. However, there are few reports on the mechanisms of peripheral blood immunity in RA.

**Methods:**

ScRNA-seq was performed on four RA samples and integrated with single-cell transcriptome data from four healthy control samples downloaded from publicly available databases for analysis.

**Results:**

A total of 52,073 cells were used for descending clustering analysis to map RA peripheral blood immune cells at single-cell resolution. Redimensional clustering analysis of four major immune cells (T cells, monocytes, B cells, and natural killer cells) revealed that double-negative T (DNT) cells were significantly altered in abundance and function. And a number of genes (including SOCS3, cAMP-responsive element modulator (CREM), B2M, MTFP1, RSRP1, and YWHAB) were specifically downregulated in DNT cells. RA T cells, especially DNT cells, exhibit significant metabolic defects and dysfunction, mainly in the form of inhibition of oxidative phosphorylation, ATP synthesis, and major histocompatibility complex (MHC)-I-mediated antigen presentation. In addition, cellular communication networks were established, and it was evident that RA is significantly attenuated in the number and intensity of cellular communication. Monocytes and T cells play key roles in the process of the immune inflammatory response through CCL and MHC-related pathways.

**Conclusions:**

This study describes the landscape of the peripheral blood immune system and cell communication in RA, characterizes the abundance of PBMCs, gene expression profiles, and changes in signaling pathways in RA patients, and identifies several key cell subpopulations (DNT and classic monocytes) and specific genes (SOCS3, CREM, B2M, MTFP1, RSRP1, and YWHAB). Meanwhile, we propose that classic monocytes in peripheral blood may migrate to sites of inflammation in synovial tissue under the chemotaxis of the chemokines CCL3 and CCL3L1, differentiate into macrophages, secrete proinflammatory cytokines, and thus participate in the inflammatory response. These findings provide new insights for the future elucidation of the peripheral blood immune mechanisms of RA and the search for new clinical therapeutic targets.

## 1. Introduction

Rheumatoid arthritis (RA) is a common antigen-mediated, multifactorial, systemic, inflammatory, chronic progressive autoimmune disease. It is characterized by chronic erosive arthritis, which manifests as a chronic inflammatory disease of the joint tissues [1–4]. RA causes the destruction of cartilage and bone tissue in the joints, ultimately leading to joint deformity and loss of mobility [5, 6]. The incidence is at least twice as high in women as in men, and the peak age of onset is between 50 and 60 years [2]. Studies have shown that RA is mainly caused by a series of immunopathological reactions triggered by the stimulation of the action of environmental factors, with infections, immune abnormalities, and genetic factors being the main factors closely associated with the pathogenesis of RA. In addition, abnormal immune system function is considered key to the development of RA [7]. Numerous drugs have been used to treat RA, but these drugs are both toxic and ineffective [8]. Therefore, there is an urgent need to elucidate the immune mechanisms that drive RA.

The persistence of autoantibodies, such as rheumatoid factor, anticitrullinated protein antibody (ACPA), and anticarbamylated protein antibody, is an important feature of RA, and these antibodies appear in the blood before the onset of disease symptoms [9–11]. Orange et al. [12] found that preinflammatory mesenchymal cells precede flares in RA patients by 1–2 weeks. They appear in the blood and subsequently invade the joint to induce synovial inflammation. Argyriou et al. [13] performed an in-depth study of CD4+ T cells in synovial fluid and peripheral blood of European patients with RA by single-cell sequencing combined with single-cell immunome library sequencing and identified two peripheral helper T-cell subsets and one cytotoxic CD4+ T-cell subset associated with RA. Wu et al. [14] constructed cellular profiles of peripheral blood and synovial tissues for different subtypes of RA patients and systematically analyzed the differences in immune status between ACPA− and ACPA+ RA patients. In conclusion, these studies suggest that the immune microenvironment of the peripheral blood is significantly altered before the onset of clinical symptoms in RA and that key cell populations and cytokines in the peripheral blood may be important in driving the development of RA. These studies have provided a preliminary understanding of the peripheral immune landscape of RA, but the study of individual cell populations is not fully developed; for example, the mechanisms of cell–cell interactions and the relationship between cells and the pathogenesis of RA are still not elucidated.

Here, we used single-cell RNA sequencing technology to analyze peripheral blood mononuclear cells (PBMCs) from RA patients and healthy individuals to characterize the composition, proportion, gene expression profile, and changes in signaling pathways of peripheral immune cells in RA patients. In contrast to previous studies, we further investigated the cellular communication of RA PBMCs to investigate the ligand-receptor pairs that play a key role in the pathogenesis. Thus, this study may help further elucidate the mechanisms of RA development and provide a theoretical basis for disease staging, the discovery of new therapeutic targets, and the identification of early diagnostic markers for this disease.

## 2. Materials and Methods

### 2.1. Sample Acquisition

According to the 2010 ACR/EULAR RA classification criteria and clinical diagnostic findings, a total of four RA patients were recruited from Xinyang, Henan Province. Detailed patient information is available in *Supplementary 1*. All RA patients were not treated with any DMARDs or immunosuppressive drugs. Meanwhile, we obtained scRNA-seq data of PBMCs from four healthy individuals from the GEO database (https://www.ncbi.nlm.nih.gov/geo/, accession number: GSE175499) [15]. The healthy control samples were also obtained from Xinyang, Henan Province. RA patients and healthy control samples are identical in terms of sample isolation and preservation methods, sequencing platforms, and reagents. The study was approved by the Institutional Review Board of Xinyang Normal University (XFEC-2021-028), and written informed consent was obtained from each patient.

### 2.2. PBMC Isolation

Peripheral blood samples from both patients and healthy individuals were isolated using the Ficoll-Hypaque density gradient centrifugation method for PBMCs isolation, and sample preparation was performed at room temperature. Cell counts were performed on each sample to determine the sample quality, the viability of all samples was greater than 90%, and cell activity and concentration of the samples met the requirements of the subsequent experiments.

### 2.3. 10× Genomics Single-Cell RNA Sequencing

Single-cell 3′-gene expression libraries were generated in the patient and healthy individual samples strictly following the protocol of the Chromium Single Cell 3′v3 Library Kit (10x Genomics). All generated libraries were high-throughput sequenced using the Illumina Nova 6000 PE150 platform. In our study, library preparation and sequencing were performed by Shanghai OE Biotech. Co., Ltd.

### 2.4. Data Preprocessing

Raw data (raw reads) of control and RA PBMCs samples generated from high-throughput sequencing were in fastq format and were demultiplexed and mapped to the human genome (build GRCh38) using CellRanger (10x Genomics, version 6.1.1). This software quantifies high-throughput single-cell RNA data by identifying barcode markers that distinguish cells in the sequence and unique molecular identifier (UMI) markers for different mRNA molecules within each cell and provides cell quality control statistics, such as number, median gene value, and sequencing saturation.

Based on the initial quality control evaluation by Cellranger, the data were further quality controlled using the Seurat software package [16]. Theoretically, most of the cells expressing the number of genes, number of UMI, and percentage of mitochondrial transcript expression will be concentrated in a certain region, so we filtered low-quality cells according to the distribution of the three indicators: nUMI, nGene, and percentage of mitochondria. The specific quality control scheme was as follows: cells with the number of genes greater than 200, the number of UMI greater than 1,000, the log_10_GenesPerUMI greater than 0.7, and the percentage of mitochondrial UMI less than 30% were retained as high-quality cells. In addition, we used DoubletFinder [17] software to check the data for potential doublets in all cells and remove them.

We then normalized the single-cell count matrix data to account for the effect of library sequencing depth. We normalize and scale the single-cell gene expression data using functions in the Seurat package. It is normalized using the “NormalizeData” function with the normalization method set to “LogNormalize.” Specifically, it is normalized by dividing the number of features per gene per cell by the total number of features per cell, multiplying by scale.factor (default 10,000), and then using log_1_p for logarithmic conversion. Then, we removed sources of nonsignificant variation by regressing the cell–cell variation in gene expression driven by batch, the number of UMIs detected, and mitochondrial gene expression, which was achieved by the “ScaleData” function. Finally, the corrected expression matrix was used as input for further analysis.

### 2.5. Dimensionality Reduction and Single-Cell Clustering

To eliminate batch effects between samples, the canonical correlation analysis method of the Seurat package was used for data integration [16]. We used the “FindIntegrationAnchors” [18] and the “IntegrateData” functions in the R toolkit, Seurat, to assess batch effects in the data and perform corrections. Highly variable genes (HVGs) were screened using the FindVariableGenes function in the Seurat package, and the expression profiles of HVGs were subjected to principal component analysis dimensionality reduction analysis. The results were visualized in two dimensions using Uniform Manifold Approximation and Projection (UMAP).

### 2.6. Cluster Marker Identification and Cell-Type Annotation

Marker gene identification was performed using the FindAllMarkers function of the Seurat package [16]. The genes that were differentially upregulated in each cell classification relative to other cell populations were the potential marker genes for each cell classification and were visualized using the VlnPlot and FeaturePlot functions. Cell types annotation was performed using the SingleR package [19], based on the public single-cell reference expression quantification public dataset in combination with the CellMarker database (http://bio-bigdata.hrbmu.edu.cn/CellMarker/) and published related literature. The correlation between the expression profile of the cells to be identified and the reference dataset was calculated. The cell type with the highest correlation in the reference dataset was assigned to the cells to be identified, eliminating to some extent the interference of human subjective factors. The identification principle is to calculate the Spearman correlation between the expression profile of each cell in the sample and the expression profile of each cell annotated in the reference dataset and to select the cell type with the highest correlation with the expression of the sample cell in the dataset as the final cell type to be identified.

### 2.7. Identification of Differentially Expressed Genes (DEGs) and Functional Enrichment Analysis

DEGs were screened using the FindMarkers function in the Seurat package [16], and differentially significant genes were screened based on a *p*-value less than 0.05 and differential multiplicity greater than 1.5-fold. Gene ontology (GO) and Kyoto Encyclopedia of Genes and Genomes (KEGG) enrichment analyses of differentially significant genes were performed using a hypergeometric distribution test.

### 2.8. Proposed Time-Series Analysis

Cell differentiation trajectories were inferred using the Monocle2 (v2.9.0) package [15]. First, the “importCDS” function of the Monocle2 package was used to convert from Seurat objects to CellDataSet objects, and the genes used to order the cells were filtered by the “differentialGeneTest” function (ordering gene, q.val < 0.01). Then, the “reduceDimension” function was used to reduce the clustering, and finally, the “orderCells” function was used to infer the differentiation trajectory.

### 2.9. Functional Enrichment Analysis

In this study, we used gene set variation analysis (GSVA). The background gene set files were first downloaded and assembled from the KEGG database (https://www.kegg.jp/) using the GSEABase package (v1.44.0). Then, individual cells were scored for pathway activity values using the GSVA package (v1.30.0) [20]. Finally, the LIMMA package (v3.38.3) was used to calculate the differences between different subgroups of signaling pathway activity. Also, GO and KEGG enrichment between the two groups was also performed by gene set enrichment analysis (GSEA) [21] using the C5 GO gene set and the C2 KEGG gene set (v7.2) from the MSigDB data (http://www.gsea-msigdb.org/gsea/msigdb). In addition, we also performed GO and KEGG analyses were performed by Metascape (https://metascape.org/gp/index.html) [22]. Partial results were visualized by using Hiplot (https://hiplot.com.cn), which is a comprehensive web platform for scientific data visualization.

### 2.10. Construction of the Protein–Protein Interaction (PPI) Network

The PPI network model was constructed using the STRING platform (https://string-db.org/) [23]. The organism was set to “*Homo sapiens*,” and the minimum protein interaction threshold was set to “low confidence (0.150)”. For the other parameters, the default settings were used to obtain the PPI network. The topological properties of the PPI network were analyzed using the network analysis function of Cytoscape software [24], and the node degree distribution and betweenness centrality of the network were calculated.

### 2.11. Cell Communication Analysis

The R package CellChat (v 1.1.3) [25] was used to analyze intercellular ligand-receptor interactions. First, the normalized expression matrix was imported, and then the CellChat object was created using the “create CellChat” function. The default parameters for the “identify Over Expressed Genes,” “identify Over Expressed Interactions,” and “project Data” functions were used for preprocessing operations. Potential ligand-receptor interactions were computed using the functions “compute CommunProb,” “filter Communication” (min.cells = 10), and “compute CommunProb Pathway.” Finally, the intercellular communication networks were aggregated using the “aggregateNet” function.

### 2.12. Statistical Analysis

Wilcoxon rank-sum test was used to detect DEGs in the scRNA-seq data, with *p*-values adjusted for false discovery rate. For the other data, statistical analysis was performed using Student's *t*-test in GraphPad Prism. Data are expressed as the mean ± SD, with statistical significance is indicated by an asterisk:  ^*∗*^*p* < 0.05,  ^*∗∗*^*p* < 0.01, and  ^*∗∗∗*^*p* < 0.001.

## 3. Results

### 3.1. Single-Cell Profiling of PBMCs in RA

**Figure 1:**
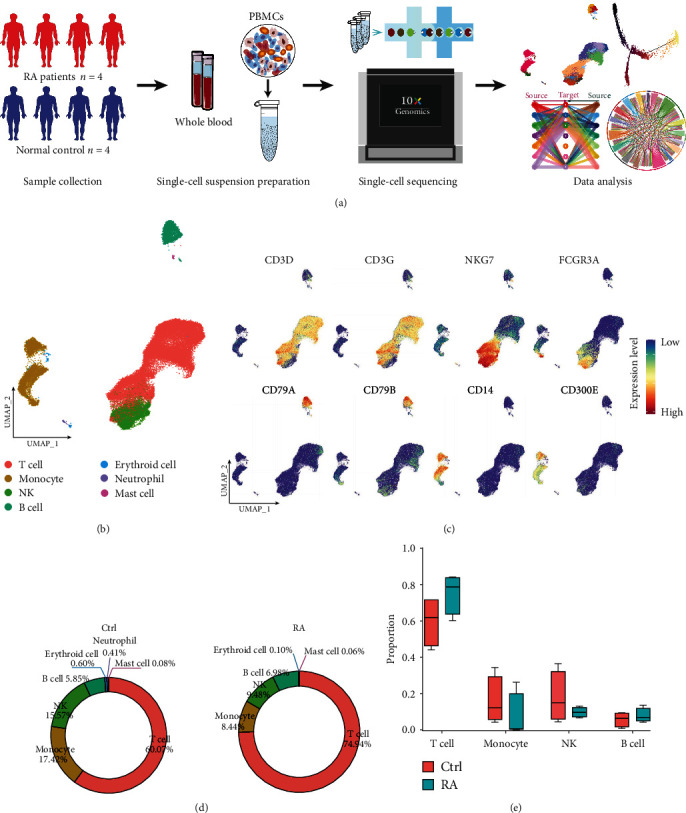
A single-cell transcriptome atlas of PBMCs in RA. (a) Ctrl and RA PBMC sample collection and processing procedures used for scRNA-seq analysis. (b) UMAP clustering distribution of 52,073 single cells. Each dot represents one cell, and each color represents the cell type. (c) Expression of marker genes in the four major immune cell types (T cells, NK cells, B cells, and monocytes). (d) Statistics of the proportion of each cell in the Ctrl and RA samples. (e) The box plot shows the change in the proportion of each cell type for the Ctrl and RA samples. Differences in the distribution of cell types between the two groups are marked with  ^*∗*^*p*values, and *p* values were calculated using a *t*-test (from left to right, *p* values are 0.1320, 0.3801, 0.3073, 0.4849, 0.0308, 0.2579, 0.9159).  ^*∗*^*p* < 0.05,  ^*∗∗*^*p* < 0.01,  ^*∗∗∗*^*p* < 0.001.

We performed an integrated analysis of single-cell transcriptome data from healthy individuals (Ctrl, *n* = 4) and RA patients (RA, *n* = 4) to characterize the single-cell profiles of PBMCs (Figure 1(a)). We briefly summarized and evaluated the data, and after removing low-quality cells using quality control indicators, such as mitochondrial gene expression, median gene number, and valid UMIs, a total of 52,073 high-quality single-cell gene expression data (including 26,294 Ctrl PBMCs and 25,779 RA PBMCs) were screen. On average, each cell contains transcript reads of 1,601 genes, with an average UMI number of 5,068 and an average number of reads of 66,649, and these data are used for subsequent analysis (*Supplementary 9*). We identified seven clusters using UMAP unsupervised clustering based on each cellular gene expression profile, combining the SingleR package, the CellMarker dataset, and known typical cell marker genes (CD3D, CD3G, NKG7, FCGR3A, CD79A, CD79B, CD14, and CD300E, etc.) (*Supplementary 2*). These cells were annotated as T cells, monocytes, natural killer (NK) cells, B cells, erythrocytes, neutrophils, and mast cells (Figures 1(b) and 1(c)). Meanwhile, after merging the data, cells from RA and Ctrl were evenly distributed in each cell group (*Supplementary 10*). The typical signature genes were specifically highly expressed in each of the major cell types, all of which have distinct gene expression patterns (*Supplementary 10*).

In addition, we observed a higher proportion of T cells, NK cells, monocytes, and B cells among all cells based on the statistical analysis of cell abundance (Figure 1(d), *Supplementary 3*). Compared with Ctrl samples, the proportion of T cells was increased in RA samples, and the proportions of monocytes and NK cells were decreased. However, no significant difference was observed in the proportions of major cell types in the PBMCs from Ctrl and RA samples (Figure 1(e)).

### 3.2. Metabolic Defects and Dysfunction of T Cells Occur in RA

**Figure 2:**
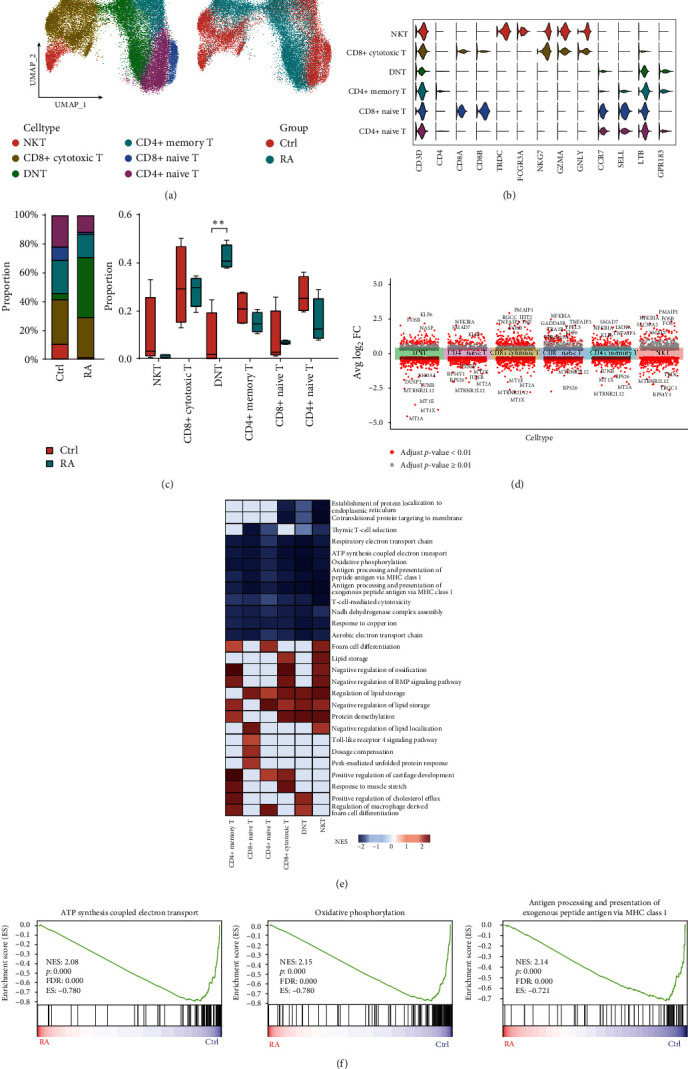
The scRNA profiles for T cells in Ctrl and RA samples. (a) UMAP clustering distribution of T cells. Each dot represents one cell. From left to right, each color represents cell type and sample type. (b) Violin plot showing the expression of major marker genes. (c) Statistical and differential change analysis of T-cell proportions in the Ctrl and RA samples (statistical analysis performed as above). (d) Differential expression analysis of T-cell subpopulations. (e, f) GSEA shows enrichment pathways in T cell subpopulations. ES, enrichment score; NES, normalized enrichment score.

First, we examined the single-cell transcriptome characteristics of the highest proportion of T cells. According to fold change >1.5 and *p*-value < 0.05, we identified 325 DEGs (RA vs. Ctrl, 210 upregulated genes and 115 downregulated genes) (*Supplementary 4*, *Supplementary 11*). GSVA revealed differences in the activity score of each cell signaling pathway. We found that pathways related to amino acid synthesis and metabolism (e.g., histidine and tryptophan metabolism, valine, leucine, and isoleucine biosynthesis, and glycine, serine, and threonine metabolism) were activated in T cells in RA, whereas oxidative phosphorylation, thermogenesis and some other pathways related to energy metabolism were inhibited (*Supplementary 11*). T cells were divided into six specific stable cell subclusters to further evaluate the changes in T-cell characteristics. Based on the expression of marker genes (CD3D, CD4, CD8D, CD8B, TRDC, FCGR3A, NKG7, GZMA, GNLY, CCR7, SELL, LTB, GPR183) in each subcluster, we defined these subpopulations as NKT cells, CD8+ cytotoxic T cells, CD4 CD8 double-negative T cells (DNT), CD4+ memory T cells, CD8+ naïve T cells and CD4+ naïve T cells (Figures 2(a) and 2(b)). We then compared the differences in the proportions of T-cell subtypes between Ctrl and RA samples. Notably, the proportion of DNT was significantly higher in the RA samples, and the proportions of the other cell subtypes were reduced in RA but not significantly different (Figure 2(c), *Supplementary 3*). To further elucidate the molecular differences between the RA and Ctrl samples, we performed a differential gene enrichment analysis and a functional enrichment analysis. A total of 674 upregulated genes and 610 downregulated genes (fold change >1.5 and adjusted *p*-value < 0.01) were found in RA T cells compared to Ctrl T cells (Figure 2(d) and *Supplementary 5*). Interestingly, the expression of metallothionein-related genes was significantly reduced among all downregulated genes in T cells. This was particularly true in DNT cells, where MT2A, MT1X, MT1E, and MT1G were significantly downregulated (Figure 2(d), *Supplementary 12*). This suggests an abnormal mitochondrial function of T cells in RA. Similarly, GSEA revealed that oxidative phosphorylation, ATP synthesis-coupled electron transport, antigen processing, and presentation of exogenous peptide antigen by major histocompatibility complex (MHC) class I were inhibited in RA T cells (Figures 2(e) and 2(f)). These results illustrate the metabolic defects and dysfunction of T cells in the disease state. In addition, we found that DNTs were significantly altered in both number and function during this process, suggesting that we can explain the mechanism of RA by alterations in DNT metabolism and explore new metabolic immune checkpoints.

### 3.3. Single-Cell Transcriptome Profiling of Monocytes

**Figure 3:**
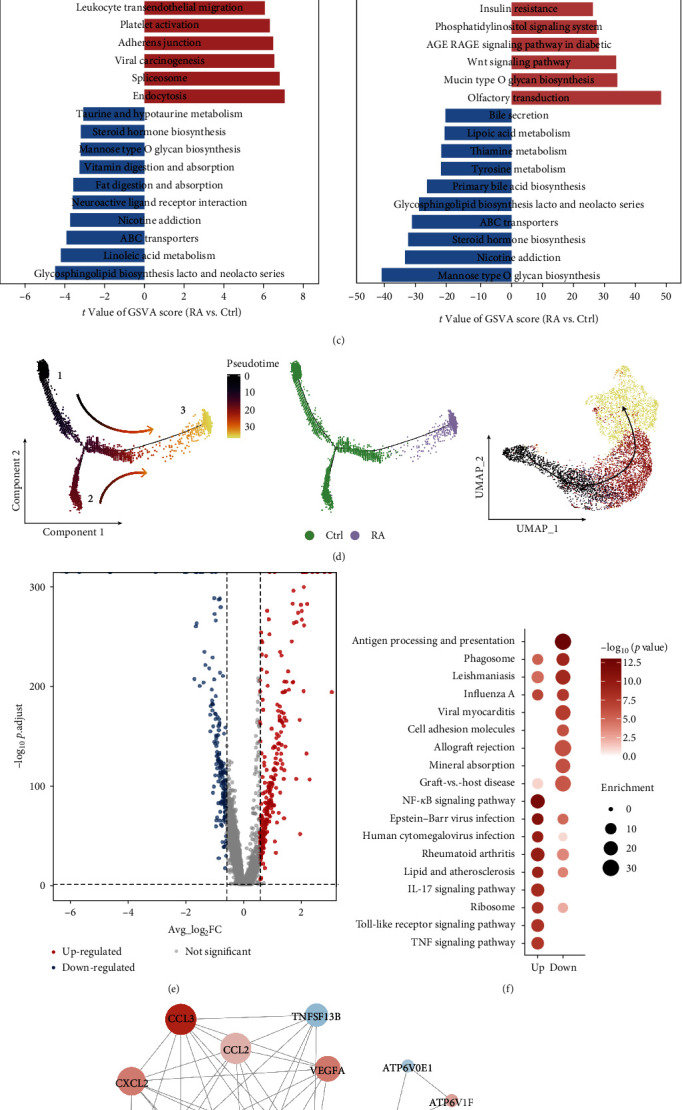
The scRNA profiles for monocytes in Ctrl and RA samples. (a) UMAP clustering distribution of monocytes. Each dot represents one cell, and each color from left to right represents cell type and sample type. (b) Expression of CD14 and CD16 on monocytes. (c) GSVA shows the difference in pathway activity scores per cell in classic monocytes and nonclassic monocytes. Each rectangle represents one pathway, with longer rectangles representing higher enrichment. The *t* values greater than 0 (red) indicate upregulated pathways, and the *t* values less than 0 (blue) indicate downregulated pathways. (d) Potential monocytes transformation trajectories inferred from Monocle2 analysis. (e) Volcano plot showing differential gene expression in monocytes at State 3 between Ctrl and RA samples. (f) KEGG analysis of DEGs in State 3. (g) Protein–protein interaction network of DEGs in the rheumatoid arthritis signaling pathway.

Monocytes are among the most important immune cells. They not only present antigens and activate self-reactive T cells but also migrate into synovial tissues to differentiate into macrophages, produce proinflammatory factors, and further transform into osteoclasts involved in joint destruction in RA patients [26]. We examined the DEGs between the RA and Ctrl samples and screened a total of 307 upregulated genes and 337 downregulated genes (fold change > 1.5 and *p*-value < 0.05) (*Supplementary 6*, *Supplementary 13*). The upregulated DEGs were mainly involved in inflammatory responses, cytokine-mediated signaling pathways, apoptotic processes, and the regulation of neutrophil chemotaxis. The downregulated DEGs were mainly enriched in type I interferon signaling pathways, interferon-gamma-mediated signaling pathways, immune system processes, and neutrophil degranulation (*Supplementary 13*). To gain insight into the molecular differences between RA and Ctrl samples, we further classified monocytes into classic monocytes (CD14+, CD16−) and nonclassic monocytes (CD14+, CD16+) based on the expression of CD14 and CD16 in the cells (Figures 3(a) and 3(b), *Supplementary 13*). Classic monocytes are mainly associated with processes, such as inflammatory response, response to cytokines, positive regulation of cytokine production, and positive regulation of cell death. Nonclassic monocytes are mainly involved in processes such as cytokines response, cellular response to cytokine stimulation, leukocyte activation, regulation of cell activation, and assembly of protein-containing complexes (*Supplementary 13*). Compared with the Ctrl samples, the proportion of classic monocytes was increased in the RA samples, and the proportion of nonclassic monocytes was correspondingly decreased. However, these changes were not significantly different (*Supplementary 3*, *Supplementary 13*). GSVA results showed that Wnt signaling pathway, ubiquitin-mediated protein hydrolysis, and phosphatidylinositol signaling system were activated in classic monocytes in RA samples, and platelet activation, leukocyte transendothelial migration, and endocrine and other factors regulating calcium reabsorption were activated in nonclassic monocytes in RA samples (Figure 3(c)).

To further explore the state of monocytes under different conditions, trajectory analysis was performed to elucidate the transcriptional transition between RA and Ctrl. State 1 was mainly composed of two types of monocytes in Ctrl samples, and State 2 was mainly composed of classic monocytes in Ctrl samples. However, State 3 showed that classic monocytes were mainly derived from Ctrl and RA samples (Figure 3(d)). Next, we performed differential expression analysis on State 3 cells and identified a total of 251 upregulated DEGs and 190 downregulated DEGs (Figure 3(e)); notably, most of these genes were also present in all monocyte DEGs (*Supplementary 13*). KEGG enrichment analysis revealed that upregulated DEGs were significantly enriched in the RA pathway (Figure 3(f)). Detailed analysis of genes enriched in RA-related pathways revealed that the expression of CCL3, CXCL8, CCL3L1, and CCL2 was upregulated in RA monocytes, while the expression of some human leukocyte antigen (HLA) genes was downregulated (Figure 3(g), *Supplementary 13*).

### 3.4. Functional and Pathway Enrichment Analysis of NK Cell and B Cell

Next, we reclustered the NK cells and further classified them into mature NK, memory NK, and immature NK based on the expression of KLRC2, PRF1, FCGR3A, and KLRC1 (*Supplementary 14*). Mature NK cells are the most abundant type in both RA and Ctrl samples (*Supplementary 3*, *Supplementary 14*). Although there was no significant difference in the proportion of cells, both groups showed unique functional enrichments. Mature NK cells in RA samples were mainly enriched in the regulation of RNA splicing, response to cytokines, and apoptosis pathways, whereas mature NK cells in Ctrl samples were mainly associated with lymphoid and leukocyte-mediated immunity, immune effector processes, and antigen processing and presentation (*Supplementary 14*). Compared to Ctrl samples, the proportion of memory NK cells was decreased, and the proportion of immature NK cells was increased in RA samples. However, these differences were not significant (*Supplementary 14*). Notably, memory NK cells in RA are more involved in processes such as cellular response to cytokine stimulation, apoptosis, and positive regulation of cytolytic processes. Immature NK cells are more enriched in processes such as chromatin organization, histone modification, and chromatin remodeling. Both memory and immature NK cells in Ctrl samples are associated with NK cell-mediated processes such as cytotoxicity, regulation of cell activation, and positive regulation of immune responses (*Supplementary 14*).

Similarly, we applied unsupervised clustering to partition all 3,337 B cells identified by UMAP. Based on the expression of CD79A, CD79B, and some other known marker genes, a total of three B-cell subtypes were identified, including naïve B cells, memory B cells, and plasma cells (*Supplementary 15*). Naïve B cells and memory B cells were the more abundant B-cell subtypes in both Ctrl and RA samples (*Supplementary 3*, *Supplementary 15*). B cells in Ctrl samples were mainly involved in processes such as phagosomes, antigen processing, presentation of exogenous peptide antigens, and lymphocyte-mediated immunity. In contrast, B cells from RA patients were more involved in pathways such as apoptosis, regulation of RNA splicing, and regulation of mRNA metabolic processes (*Supplementary 15*).

### 3.5. Reduced Intercellular Communication in PBMCs of Patients with RA

Cellular interactions between immune cells play a key role in the cellular activation that ultimately leads to the development of disease symptoms in RA patients. We then constructed a cell–cell interaction map by correlating ligands with their corresponding receptors. This map depicts all altered interactions in RA samples compared to normal samples. In Ctrl samples, we identified 96 significant ligand-receptor pairs in 14 cell subpopulations that were distributed across 41 signaling pathways, including the MHC-I, MIF, CLEC, MHC-II, CD99, GALECTIN, ITGB2, RESISTIN, CD22, and CD45 pathways (*Supplementary 16*). Seventy-one significant ligand-receptor pairs were identified in RA samples and were distributed in 29 pathways, including the MIF, MHC-I, CLEC, CD99, ADGRE5, GALECTIN, MHC-II, THBS, ICAM, ANNEXIN, CD22, and CD45 pathways (*Supplementary 18*). We identified four groups of signaling pathways based on structural similarity and functional similarity, respectively (*Supplementary 16*, *Supplementary 18*).

**Figure 4:**
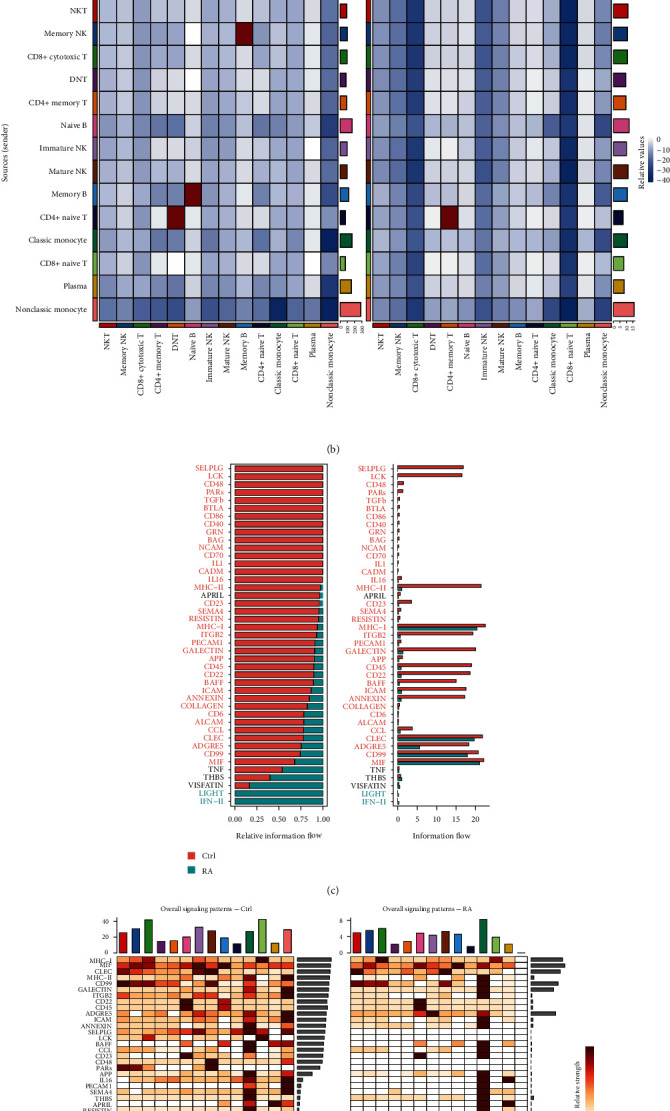
Comparative analysis of cell communication networks in Ctrl and RA samples. (a) Comparison of the number and strength of interactions of cellular communication networks of cells in Ctrl and RA PBMCs. (b) Heatmap showing the difference in intensity and number of interactions of different cells in different signals in PBMCs of Ctrl and RA samples. The top colored bar indicates the sum of the column values shown in the afferent pathways heatmap. The colored bar on the right indicates the sum of the outgoing signals. (c) The rank of all significant pathways is based on the difference in total information flow in the inferred network between the Ctrl and RA samples. The total information flow of the signaling network is calculated by summing the probability of all communications in the network. The top pathway colored in red is more common in the Ctrl samples, and the bottom pathway colored in green is more common in the RA samples. (d) Heatmap comparison of changes in afferent or efferent pathways associated with each cell population in the Ctrl and RA samples.

Comparing the interaction networks of the two groups, we found that the total number of interactions between different cells was reduced, and the intensity of the interactions was attenuated in the RA samples compared to the Ctrl samples (Figure 4(a)). Furthermore, both the number and intensity of interactions between CD4+ memory T cells and CD4+ naïve T cells were increased. The number of interactions between memory B cells and naïve B cells, and memory NK cells was increased, but the intensity was decreased (Figure 4(b)). In Ctrl samples, CD4+ naïve T cells were the major receiving cell population, and nonclassic monocytes were the major sending cell population (*Supplementary 16*). In RA samples, both the receiving and the sending signals were dominated by classic monocytes (*Supplementary 18*). Next, we identified conserved and environment-specific pathways by comparing the information flow of each pathway enriched by cell population interactions. We ranked the important pathways based on the difference in total information flow in the inferred network between Ctrl and RA samples. The MHC-I, CLEC, and MIF pathways were the major enriched pathways identified in Ctrl samples, and the IFN-II and LIGHT pathways were the major enriched pathways identified in RA samples (Figures 4(c) and 4(d), *Supplementary 17*, *Supplementary 19*). In addition, we identified a total of 15 upregulated signaling ligand-receptor pairs (including CCL3-CCR1, CCL3L1-CCR1, IFNG-IFNGR1:IFNGR2, TNF-TNFRSF1B, etc.) (*Supplementary 7*) and 68 downregulated signaling ligand-receptor pairs (including CCL5-CCR1, MIF-CD74:CXCR4, MIF-CD74:CD44, IL16-CD4, etc.) (*Supplementary 8*) based on differential gene expression analysis by comparing ligand-receptor pairs and the communication probabilities between RA and Ctrl samples for each cell group pair. These results indicated that the intercellular communication in the PBMCs of RA patients was significantly decreased.

### 3.6. Construction of a T Cell and Monocyte-Based Regulatory Network for RA

**Figure 5:**
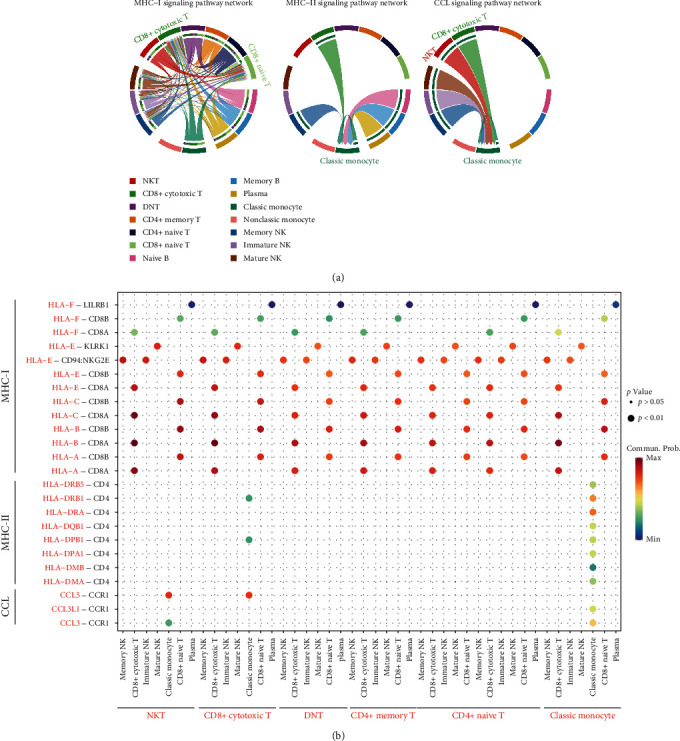
A T-cell and monocyte-based regulatory network for RA. (a) Chord diagram showing the CCL, MHC-I, and MHC-II signaling pathway network. (b) Bubble plots showing ligand-receptor pairs of the CCL, MHC-I, and MHC-II pathway network.

Notably, T cells and monocytes interacted most strongly with other cell types. Combined with the results of functional enrichment analysis and cellular abundance, we constructed a T-cell and monocyte-based RA regulatory network. As shown in Figure 5, we further analyzed the interacting ligand-receptor pairs in CCL- and MHC-related pathways. In the MHC-I pathway, CD8+ T cells act as the major receptor cells through CD8A and CD8B binding to ligand proteins from other cells with HLA class I proteins and HLA class II proteins. As described in previous results, HLA class I proteins interact with SOCS3, B2M, and YWHAB, and HLA class II proteins interact with CC and CXC family chemokines in monocytes. In contrast, in the MHC-II pathway, classic monocytes function mainly through their cell surface CD4 receptor proteins in combination with HLA-DRB1 and HLA-DPB1 from CD8+ cytotoxic T cells and HLA family proteins from themselves. In addition, as receptor cells, classic monocytes express relatively high levels of CCR1, which regulates the CCL pathway by binding to CCL3, CCL5, and CCL3L1 of NK cells, NKT cells, CD8+ cytotoxic T cells, and themselves. Taken together, our results predict that T cells and monocytes have a critical role in the development of RA through antigen presentation and CCL-related pathways.

### 3.7. SOCS3, B2M, and YWHAB are Significantly Downregulated Specifically in DNT Cells

**Figure 6:**
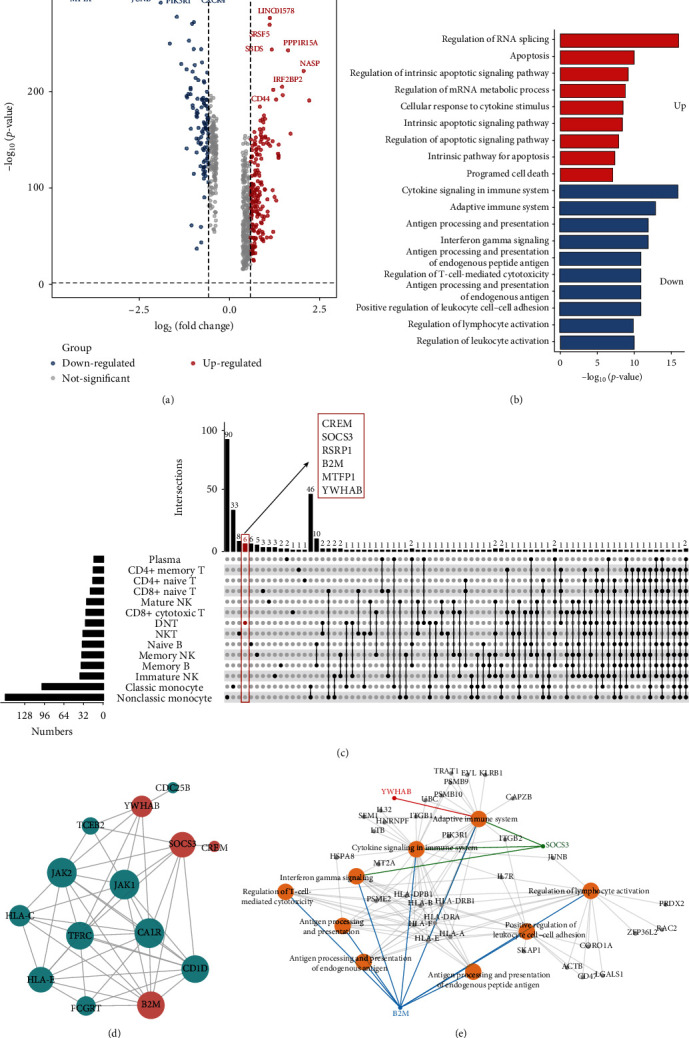
(a) Volcano plot showing the DEGs of DNT cells between Ctrl and RA patients. (b) Functional enrichment analysis of the DEGs. (c) Upset plot showing the integrated comparative analysis of downregulated DEGs in the major immune cells between Ctrl and RA groups. (d) PPI network of six specific downregulated genes in DNT cells. (e) Gene function network diagram of downregulated DEGs.

Previously, we further subdivided the four major immune cells (T cells, monocytes, B cells, and NK cells) into 14 cell subpopulations. Statistical analysis of the abundance of these cell subpopulations revealed that DNT cells were more predominant and significantly increased in RA samples. However, the downregulated DEGs showed a more pronounced functional enrichment. The functional and gene expression characteristics of DNT cells were, therefore, further investigated. By differential expression analysis, a total of 190 genes were upregulated and, 112 genes were downregulated (Fold Change > 1.5, *p*-value < 0.05) (Figure 6(a)). Furthermore, the upregulated genes are mainly associated with the regulation of RNA splicing, apoptosis, and regulation of the mRNA metabolism process. While the downregulated genes are mainly involved in processes such as cytokine signaling in the immune system, the adaptive immune system, antigen processing and presentation, and positive regulation of leukocyte cell–cell adhesion (Figure 6(b)). Comparing with other cells, we identified six genes specifically downregulated in DNT cells (log_2_FC < −1, *p*-value < 0.05), namely cAMP-responsive element modulator (CREM), SOCS3, RSRP1, B2M, MTFP1, and YWHAB (Figure 6(c), *Supplementary 20*). Notably, CREM, SOCS3, RSRP1, and MTFP1 were upregulated in PBMCs of RA samples but significantly downregulated in the DNT cells of Ctrl samples (*Supplementary 20*). PPI network analysis of these genes and their related genes revealed direct or indirect interactions among SOCS3, B2M, YWHAB, and CREM (Figure 6(d)). Among them, SOCS3, B2M, and YWHAB also interact with the JAK family and the HLA family. In addition, SOCS3, B2M, and YWHAB are involved in some critical processes, such as adaptive immune system, antigen processing and presentation, interferon-gamma signaling, and regulation of leukocyte activation (Figure 6(e)). Cell communication analysis revealed that in RA patients, DNT cells interact with T cells, B cells, NK cells, and classic monocytes mainly through MIF, MHC-I, CLEC, THBS, GALECTIN, CD22, and ADGRE5 pathways (*Supplementary 20*). In RA patients, the probability of ligand-receptor-to-regulatory communication of the relevant pathways was reduced in all cases (*Supplementary 21*). Taken together, these results suggest that despite the low percentage of DNT cells, they may play an essential role in disease pathogenesis.

## 4. Discussion

RA is a common chronic inflammatory autoimmune disease affecting approximately 1% of the world's population. Its etiology is linked to a variety of factors, including environmental, genetic, autoimmune, and more. But no matter how advanced the treatments and tools, there is no cure for RA, which causes pain, disability, and emotional, social, and economic challenges. The etiology of RA is not fully understood. In the clinical setting, the immune response plays an important role; peripheral blood is the main channel of the immune system, and PBMCs are the immune cells that initiate the autoimmune inflammatory process [27]. Previous studies have reported that peripheral blood immune cell subsets are severely dysregulated in number and function in RA patients [28], but the mechanisms of peripheral immunity in RA remain unclear. Therefore, a comprehensive investigation of the peripheral blood immune cell profile in RA is essential to elucidate the peripheral immune mechanisms of RA.

Using scRNA-seq, we described the immune landscape and systematically characterized the cellular and molecular characteristics of the major immune cells (T cells, monocytes, B cells, and NK cells) in the peripheral blood of RA and normal individuals. Although there are relevant studies on scRNA-seq of RA PBMCs, they differ from our study, especially geographical differences and clinical typing of patients. We performed a dimensional clustering analysis of these four immune cells, identified a DNT cell that was significantly altered in abundance and function, and captured a set of significantly altered genes that may serve as potential targets in RA. This study also revealed that T cells in RA undergo metabolic defects and dysfunction, mainly in the form of inhibition of oxidative phosphorylation, ATP synthesis, and MHC-I-mediated antigen presentation. In addition, cell communication analysis further explored the major roles played by these immune cells in the cell communication network, the synergistic effects between cells, and the dynamic changes of cell communication under different conditions, and we found that RA is significantly weakened in the number and intensity of cell communication, in which monocytes and T cells play an important role. These results suggest that T cells and monocytes are significantly altered at the single-cell transcriptomic level during the development of RA.

As a major player in the inflammatory process, T cells play a key role in the pathogenesis of RA. It has been reported that T cells are dysfunctional and metabolically defective in RA patients [29–32]. In our work, we filtered out low-quality and low-viability cells based on the percentage of mitochondrial genes, and we found in our results that the expression of metallothionein-related genes was significantly downregulated in T cells. Metallothioneins are involved in the regulation of mitochondrial pathophysiological processes through various pathways, such as mitochondrial redox, respiratory chain electron transfer, apoptotic signaling, enzyme activity, metal ions, membrane transition pore, mitochondrial DNA, and mitochondrial production, and have apparent protective effects against mitochondrial dysfunction [33]. These features suggest the possible presence of abnormal mitochondrial function in T cells in the peripheral blood of RA patients, which is consistent with previous studies. Mitochondria are sites of energy production involved in calcium homeostasis, lipid synthesis, apoptosis, and cell cycle processes. They are major producers of reactive oxygen species and metabolic intermediates produced in the TCA cycle [34]. It has been shown that metabolic abnormalities within the T cells of RA patients that disrupt reactive oxygen species signaling cause T-cell overproliferation and contribute to the conversion of T cells into RA-causing proinflammatory T cells. Thus, they drive the initiation and development of arthritis and autoimmune responses [35–37]. Cornelia [38] found that T cells from RA patients have an impaired TCA cycle due to the absence of the mitochondrial protein SUCLG2, which leads to the accumulation of excess acetyl coenzyme A and causes acetylation of the microtubule system, ultimately promoting the migratory behavior of T cells and disrupting the body's immune tolerance. Conversely, inhibition of microtubule acetylation in patient T cells attenuated cell migration, and further experiments confirmed that it had histoprotective effects against synovial inflammation in a humanized mouse model. Again, it was confirmed that abnormal mitochondrial metabolism in T cells is closely associated with the development of RA.

In addition, the results of GSVA showed that some amino acid metabolism (histidine, tryptophan, glycine, serine, and threonine) and amino acid synthesis (leucine and isoleucine) related pathways were enhanced in RA patients. Both threonine and tryptophan are involved in the regulation of cellular stress responses. Also, they affect the metabolism of interleukins, tumor necrosis factors (TNF), and lipopolysaccharides by participating in pathways such as protein phosphorylation and lipoprotein glycosylation [39]. Tryptophan metabolism has also been reported to be closely related to the defense mechanisms of the inflammatory process in RA [40]. In addition, abnormal leucine and glycine metabolism may lead to immune imbalance in RA patients and accelerate RA disease progression [41, 42]. Weyand found that T cells in RA have defective mitochondria by studying cellular and mouse models, and further screening of T-cell mitochondrial products revealed that T cells in RA are deficient in aspartate [30]. A decrease in aspartate, a messenger molecule between mitochondria and the endoplasmic reticulum, leads to an endoplasmic reticulum stress response and consequently overproduction of TNF, resulting in a metabolic defect that renders T cells highly efficient proinflammatory effector cells [31, 32]. In summary, T cells in RA show abnormalities in glucose metabolism, lipid metabolism, mitochondrial metabolism, and amino acid metabolism, suggesting that we can study the metabolic checkpoints of T cells and reveal the mechanism of RA from the alteration of T-cell metabolism to provide a reference for RA auxiliary diagnosis and treatment.

In contrast to previous studies, we identified a class of cells, DNT cells, among the T cells that were not only functionally significantly attenuated but also had a significantly increased proportion of cells. We speculate that DNT cells may play a key role in the initiation of the autoimmune inflammatory process and that the increase in their proportion may be compensatory to compensate for their functional deficiency. Of course, this speculation requires further experiments to confirm. The presence of small amounts of CD4−CD8− T cells has been reported in synovial tissue [14], possibly from peripheral blood. The ability of DNT not only to escape activation-induced cell death but also to be one of the major sources of pathogenic cytokines, such as IL-17, suggests to us that DNT may be at the root of the difficulty in eradicating autoimmune diseases [43, 44]. The expansion of DNT cells and the demonstration of pathogenic or regulatory effects have been observed in patients with autoimmune diseases, including systemic lupus erythematosus [45, 46], autoimmune lymphoproliferative syndrome [47], and Sjögren's syndrome [48, 49]. However, reports on the mechanism of DNT action in RA are rare. In addition, studies have evaluated the feasibility, safety, and efficacy of allogeneic DNT as a CAR T-cell therapy platform, confirming that DNT is a promising universal T-cell agent for the treatment of multiple clinical tumors [50]. Therefore, it is feasible to investigate the mechanism of action of DNT in RA, explore new therapeutic targets, and design drugs and therapeutic regimens for them.

By comparing the DEGs in each cell type, we identified six genes that were specifically downregulated in DNT cells: SOCS3, CREM, B2M, MTFP1, RSRP1, and YWHAB. Among them, we found that CREM expression was upregulated in peripheral blood. Targeted bisulphite sequencing and reverse transcription-PCR experiments revealed that the CREM promoter was hypomethylated, and the expression of CREM was upregulated in RA [51]. The CREM is a cAMP-controlled transcription factor closely associated with the regulation of the immune system. CREM is involved in the pathogenesis of systemic lupus erythematosus (SLE) [52, 53], in addition to various immune-mediated validation processes. In particular, it affects many target genes in T cells (IL-2, IL-17, IL-21, and the TH2 cytokines IL-4 and IL-13) through transcriptional and epigenetic regulation [54, 55]. Most of these potential target genes have been implicated in the pathogenesis of arthritis [55, 56]. CREM plays a critical role in the metabolism, function, and fate of T cells [57]. It has been shown that T cells in SLE patients have a reduced ability to produce IL-2 in response to antigenic stimuli, leading to their susceptibility to viral and bacterial infections [58]. In patients, CREM is the main reason for the downregulation of IL-2 expression in T cells [59, 60]. Similarly, we found that SOCS3 expression was upregulated in the whole blood of RA patients [61], which is consistent with previously reported results. However, we found that SOCS3 expression was downregulated in DNT cells. The main reason for this result may be the different sequencing units of bulk RNA-seq and scRNA-seq. SOCS3, a cytokine signaling inhibitor, is involved in the regulation of inflammatory processes and activation of JAK/STAT signaling, and SOCS3 is involved in the regulation of inflammation in RA through the cholinergic anti-inflammatory pathway [62]. Induction of its expression can reduce synovial inflammation [63], but its regulatory role in RA peripheral blood has not been confirmed. Overall, the role of these key genes in RA has been reported to a greater or lesser extent, but their specific mechanisms of action still need to be investigated in more detail. In our study, we found that RA T cells were dysfunctional, mainly manifested by inhibition of the antigen presentation pathway of MHC class I molecules. B2M, a component of MHC class I, is involved in the regulation of HLA class I molecules and is significantly downregulated in DNT cells, which may contribute to the defective class I antigen presentation. In addition, we found interactions among YWHAB, SOCS3, B2M, and CREM, suggesting that these genes may be important contributors to T-cell dysfunction and metabolic abnormalities and may serve as potential therapeutic targets for RA.

**Figure 7:**
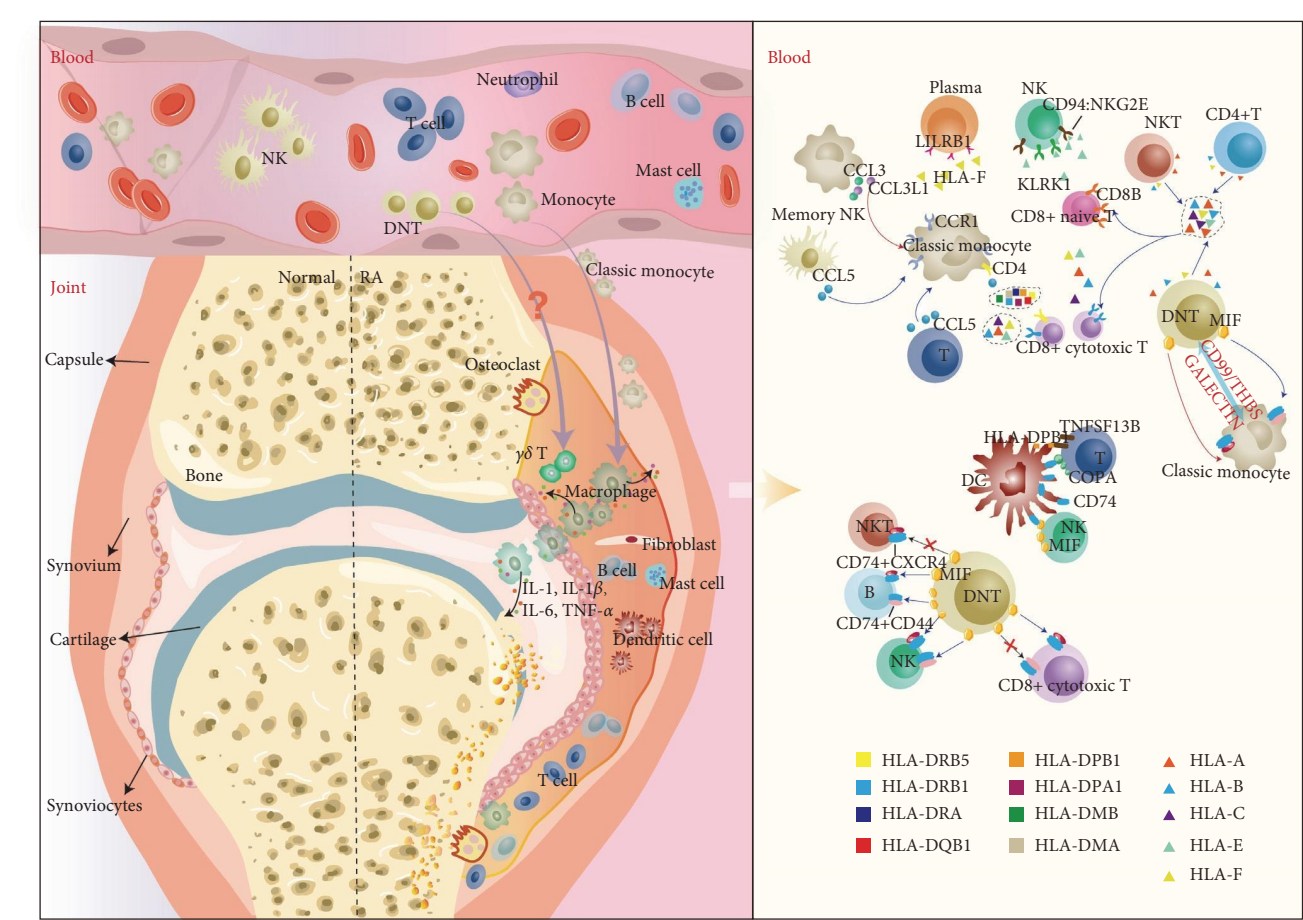
A model of the changes in the cellular interactions of the blood in RA. Red arrows indicate upregulated expression, and blue arrows indicate downregulated expression.

Furthermore, we constructed intercellular communication networks for PBMCs from healthy individuals and RA patients separately. Both the number and strength of intercellular interactions were significantly reduced in PBMCs from RA patients. And we identified key cell populations that may drive disease pathogenesis as well as important signaling changes. Studies have shown that peripheral blood-derived classic monocytes can differentiate into macrophages and infiltrate the synovial tissue or joint fluid of RA patients and that prolonged activation of macrophages promotes inflammatory responses. Combined with existing studies, we constructed a model of the changes in cellular interactions of blood in RA (Figure 7) [12, 14, 64].

Macrophage CCL3 expression was significantly upregulated in RA synovial tissue, but CCR1 expression was absent. In the peripheral blood of RA patients, classic monocyte CCL3 and CCL3L1 expression was significantly upregulated, with no significant changes in CCR1 expression levels. As shown in Figure 7, peripheral blood NK cells and T cells interacted with classic monocytes via CCL5-CCR1, and classic monocytes interacted with each other via CCL3L1-CCR1 and CCL3-CCR1, and our interaction results showed that the communication intensity of the former was decreased and the communication probability of the latter was increased. In addition, the classic monocytes in peripheral blood interacted with other cells through the MHC-II pathway, and the communication intensity of this pathway was attenuated in RA. The expression of HLA class II genes, the major ligand in the MHC-II pathway, was significantly downregulated in classic monocytes in RA, and there was a relationship between this class of genes and inflammatory factors such as TNF and IL1B, and chemokines such as CCL3 and CCL3L1, and chemokines such as CCL3 and CCL3L1.

**Figure 8:**
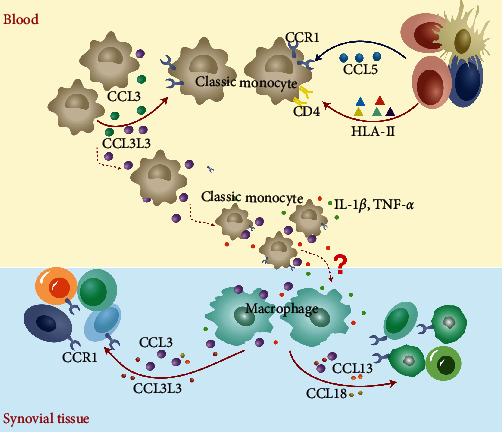
Mechanism of migration and differentiation of classic monocytes from peripheral blood to synovial tissue in RA patients.

Prior to the development of synovitis in RA patients, the autoimmune tolerance outside the joint is disrupted, and the autoimmune process is initiated, during which some cells or cytokines from the peripheral blood infiltrate into the synovial tissue or joint fluid and promote the inflammatory response. Whether this process is associated with CCL3 and CCL3L1 is still unclear. Based on these results, we hypothesize that classic monocytes in peripheral blood may migrate to sites of inflammation in synovial tissue under the chemokines CCL3 and CCL3L1, differentiate into macrophages, secrete proinflammatory cytokines, and thus participate in the inflammatory response (Figure 8). We will also follow-up with further research on this hypothesis.

Our study also has some limitations and shortcomings. On the one hand, our limited sample size may cause some bias in the results. On the other hand, our study still needs more adequate functional experimental validation. However, through this study, we also identified some cell subpopulations (DNT, classic monocytes) and genes (SOCS3, CREM, B2M, MTFP1, RSRP1, and YWHAB) of research significance. Meanwhile, we propose that classic monocytes in peripheral blood may migrate to sites of inflammation in synovial tissue under the chemotaxis of the chemokines CCL3 and CCL3L1, differentiate into macrophages, secrete proinflammatory cytokines, and thus participate in the inflammatory response. We believe that our work will contribute to the understanding of the peripheral immune landscape of RA patients and provide a valuable resource for future in-depth exploration of the pathogenesis of RA and the search for potential therapeutic targets.

## Data Availability

The dataset of healthy controls in this study is available in the online repository. These data can be found in the GEO database (https://www.ncbi.nlm.nih.gov/geo/, accession number: GSE175499) and the 10x Genomic Database (https://www.10xgenomics.com/resources/datasets/). Sequence data of RA patients have been uploaded to the National Genomic Data Center (https://ngdc.cncb.ac.cn/) under the accession number HRA003364.

## Supplementary Materials

Supplementary 1 Clinical characteristics of samples utilized.

Supplementary 2 Marker genes of all clusters.

Supplementary 3 Total number of cells per celltype.

Supplementary 4 T-cell DEGs.

Supplementary 5 T-cell DEGs by subcell type.

Supplementary 6 Monocyte DEGs.

Supplementary 7 Ligand receptor pairs that are upregulated in RA patients

Supplementary 8 Ligand receptor pairs that are downregulated in RA patients.

Supplementary 9 Quality control of single-cell RNA sequencing.

Supplementary 10 The scRNA profiles for PBMCs of Ctrl and RA.

Supplementary 11 Differences in pathway activity scores for each cell are shown by GSVA analysis. Each rectangle represents one pathway, longer rectangles represent higher enrichment. The *t* values greater than 0 (blue) indicate upregulated pathways, and the *t* values less than 0 (green) indicate downregulated pathways.

Supplementary 12 Top 10 DEGs in RA PBMCs DNT cells. Violin plot showing the top 10 DEGs between the Ctrl and RA samples. The y-axis indicates log-scaled normalized counts (*p* values were calculated by the Wilcoxon rank sum test,  ^*∗*^*p* < 0.05,  ^*∗∗*^*p* < 0.01, and  ^*∗∗∗*^*p* < 0.001).

Supplementary 13 Differential analysis of monocyte genes and functions in PBMCs.

Supplementary 14 The scRNA profiles for NK cells in Ctrl and RA samples.

Supplementary 15 The scRNA profiles for B cells in Ctrl and RA samples.

Supplementary 16 Communication networks between cell subpopulations in the PBMCs of Ctrl samples.

Supplementary 17 Network analysis of the MHC-I pathway in PBMCs from Ctrl samples.

Supplementary 18 Communication networks between cell subpopulations in the PBMCs of RA patients.

Supplementary 19 Network analysis of the MIF pathway in PBMCs from RA samples.

Supplementary 20 Analysis of DEGs and signaling pathways in DNT cells.

Supplementary 21 Dot plot showing the changing signaling ligand-receptor pairs between DNT cells and other cells.
