# Supplementary figures and images for: Analyzation of the Peripheral Blood Mononuclear Cells Atlas and Cell Communication of Rheumatoid Arthritis Patients Based on Single-Cell RNA-Seq
Source: J Immunol Res. 2023 Aug 12;2023:6300633. doi: 10.1155/2023/6300633 (PMC10439836; doi:10.1155/2023/6300633)

A

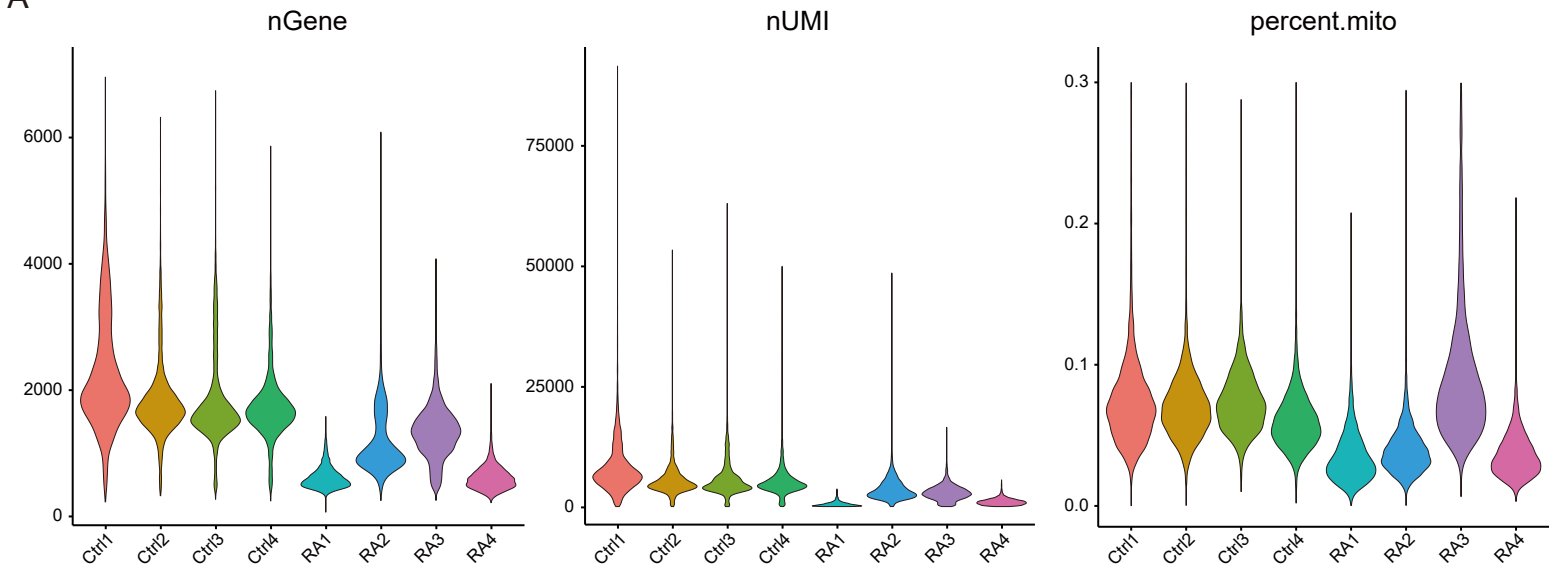

B

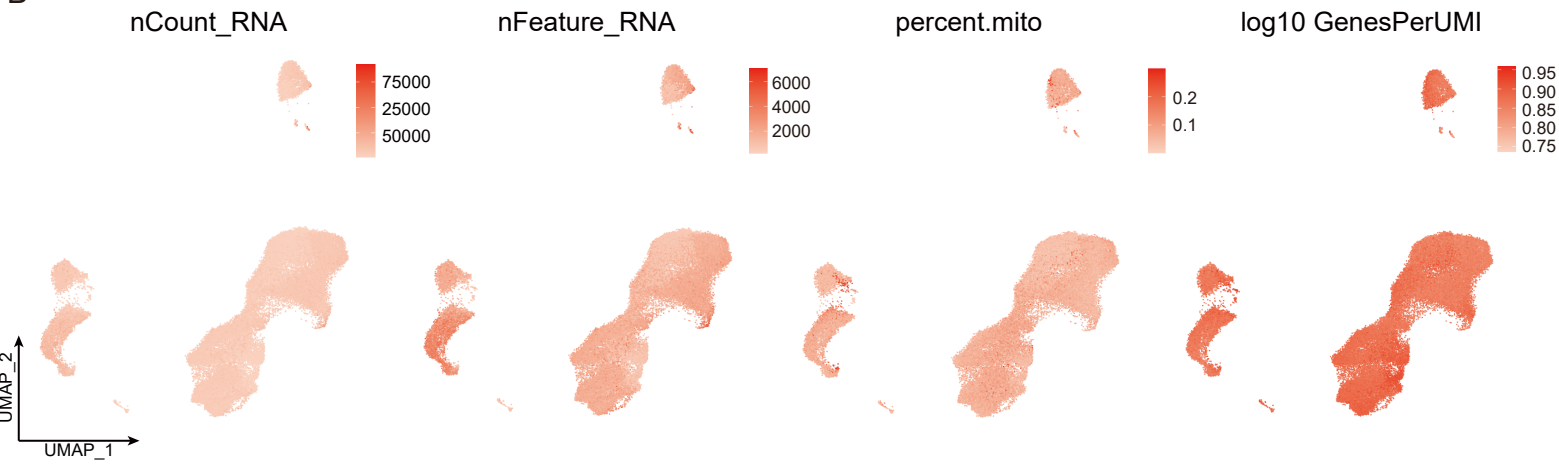

Supplement: Supplementary 9 — Quality control of single-cell RNA sequencing. [file 6300633.f9.pdf]

A

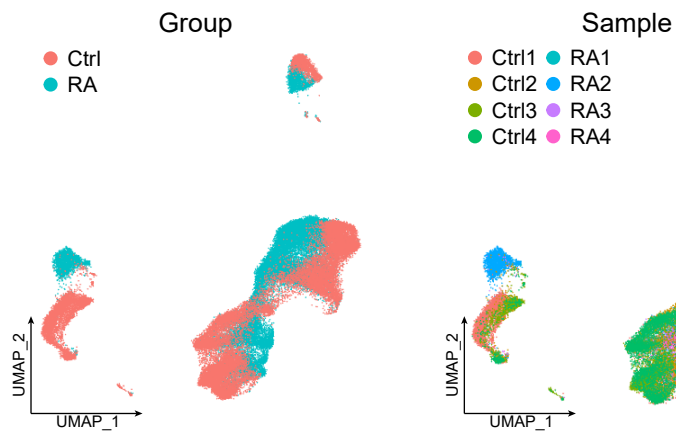

B

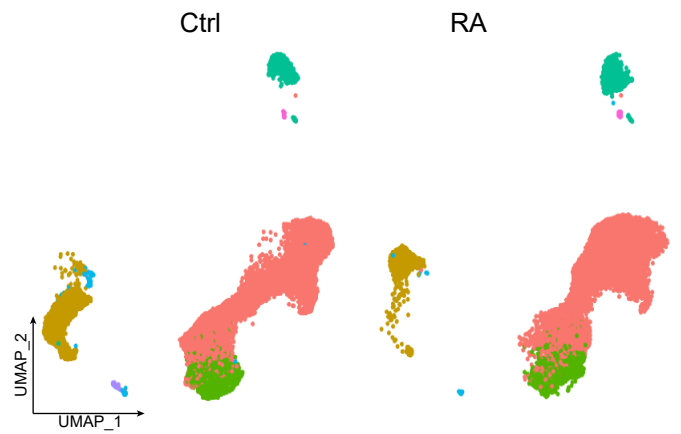

C

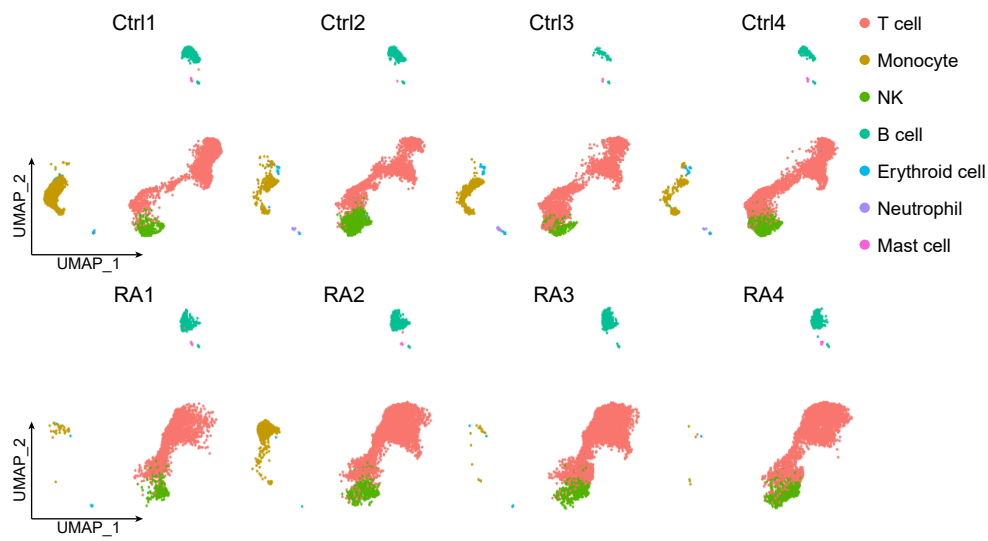

D

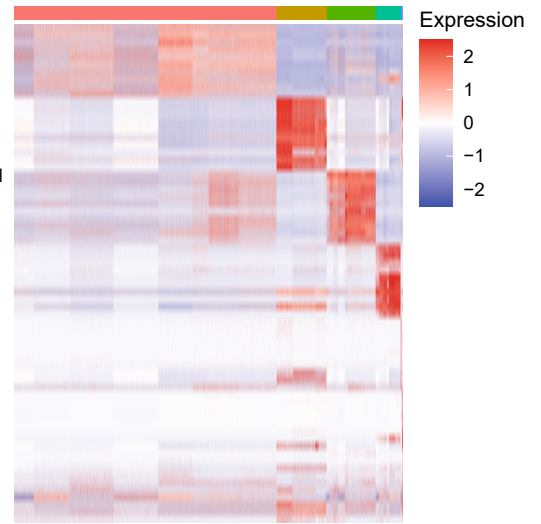

Supplement: Supplementary 10 — The scRNA profiles for PBMCs of Ctrl and RA. [file 6300633.f10.pdf]

# T cell

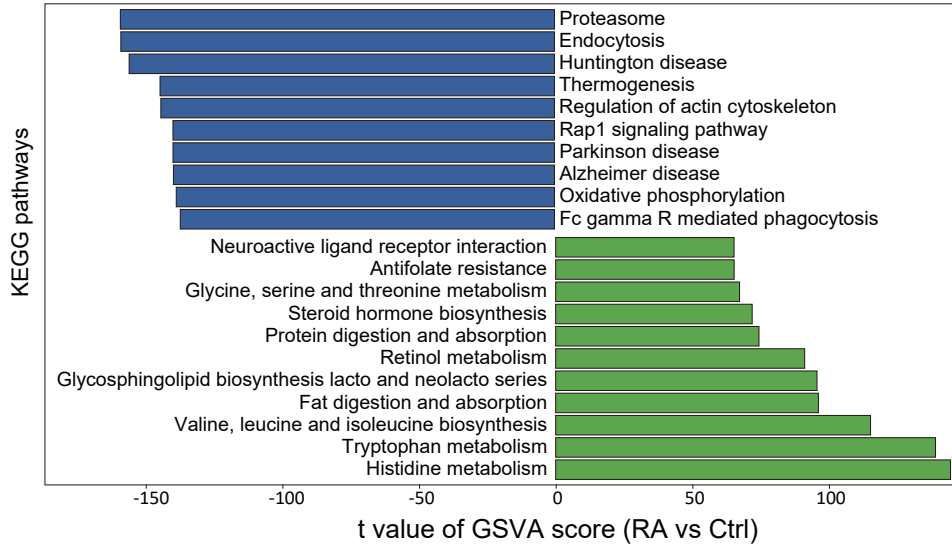

Supplement: Supplementary 11 — Differences in pathway activity scores for each cell are shown by GSVA analysis. Each rectangle represents one pathway, longer rectangles represent higher enrichment. The t values greater than 0 (blue) indicate upregulated pathways, and the t values less than 0 (green) indicate downregulated pathways. [file 6300633.f11.pdf]

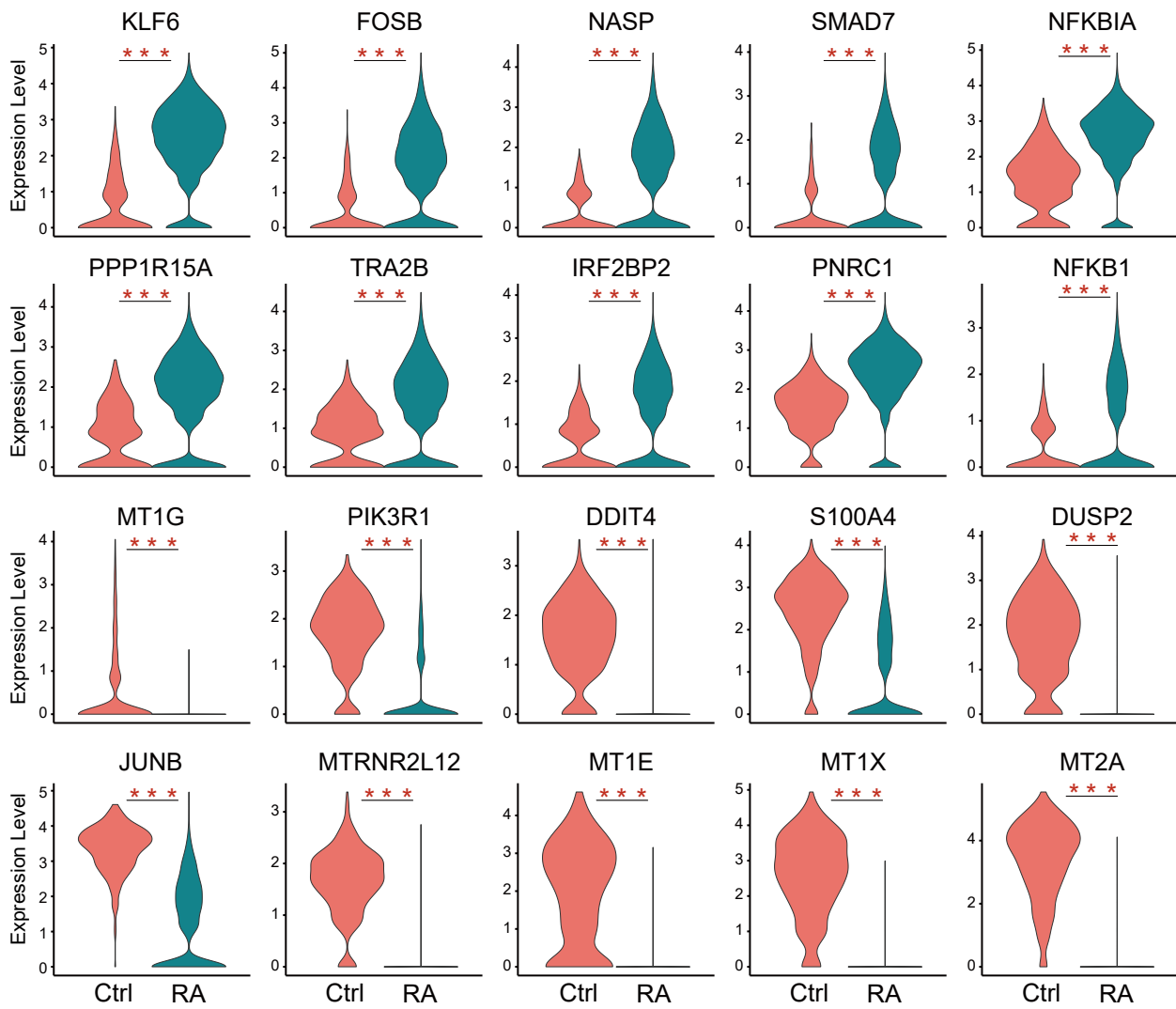

Supplement: Supplementary 12 — Top 10 DEGs in RA PBMCs DNT cells. Violin plot showing the top 10 DEGs between the Ctrl and RA samples. The y-axis indicates log-scaled normalized counts (p values were calculated by the Wilcoxon rank sum test, ∗p < 0.05, ∗∗p < 0.01, and ∗∗∗p < 0.001). [file 6300633.f12.pdf]

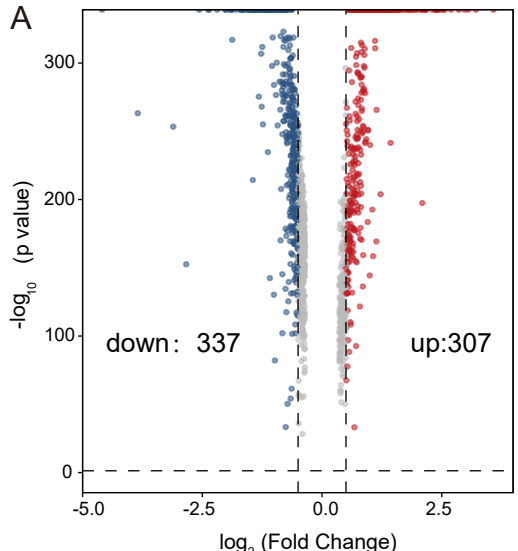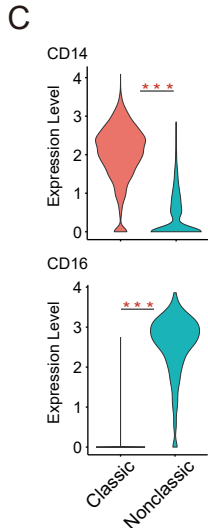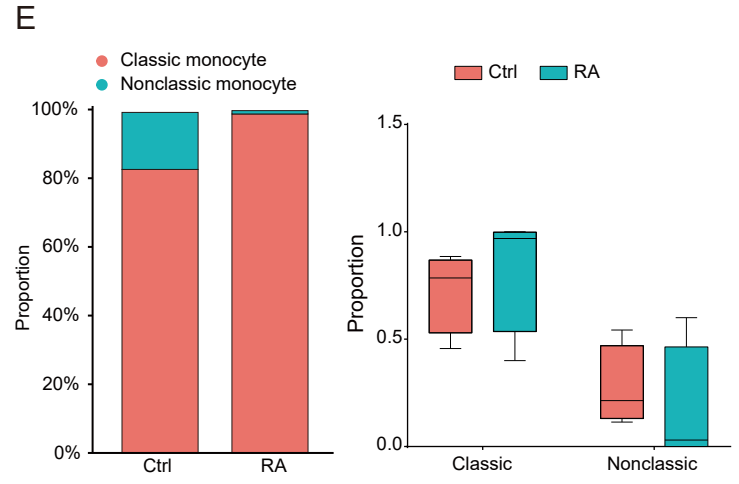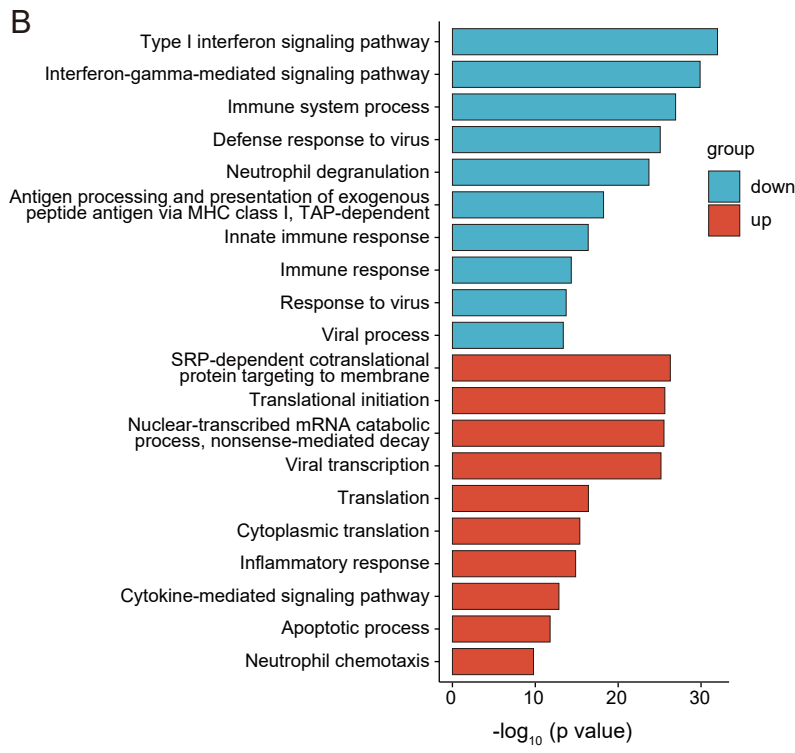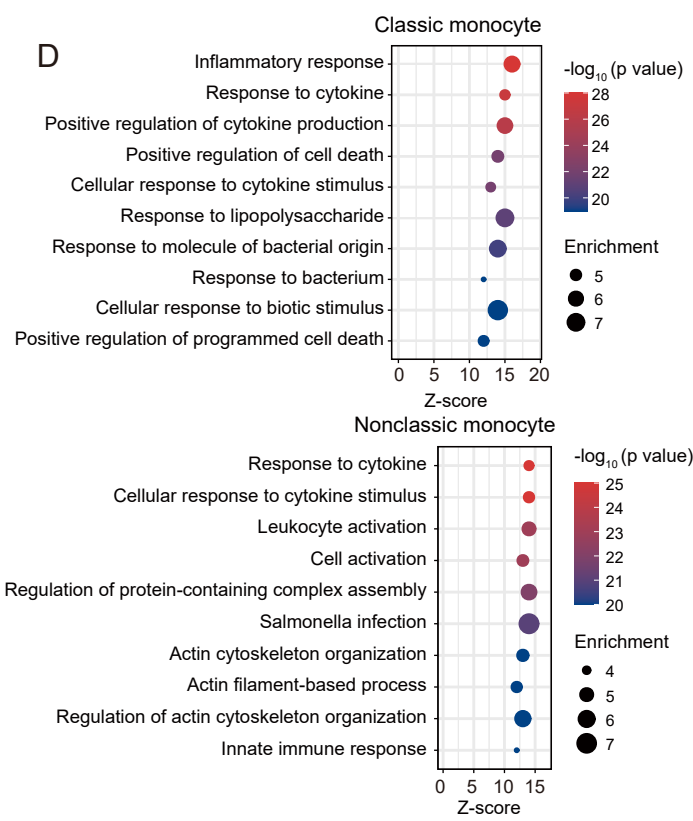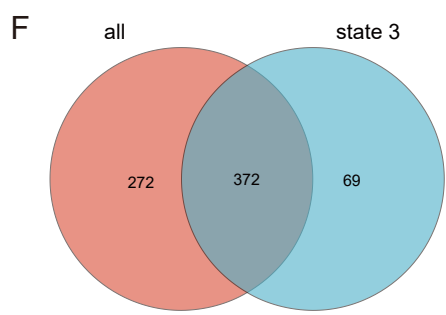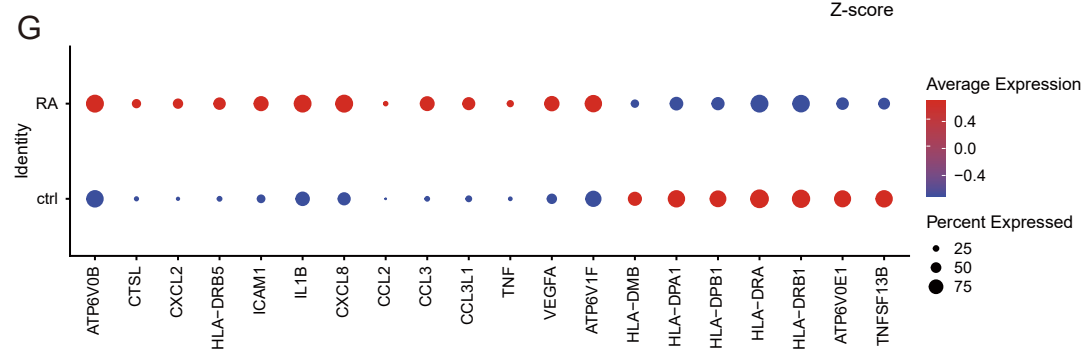

Supplement: Supplementary 13 — Differential analysis of monocyte genes and functions in PBMCs. [file 6300633.f13.pdf]

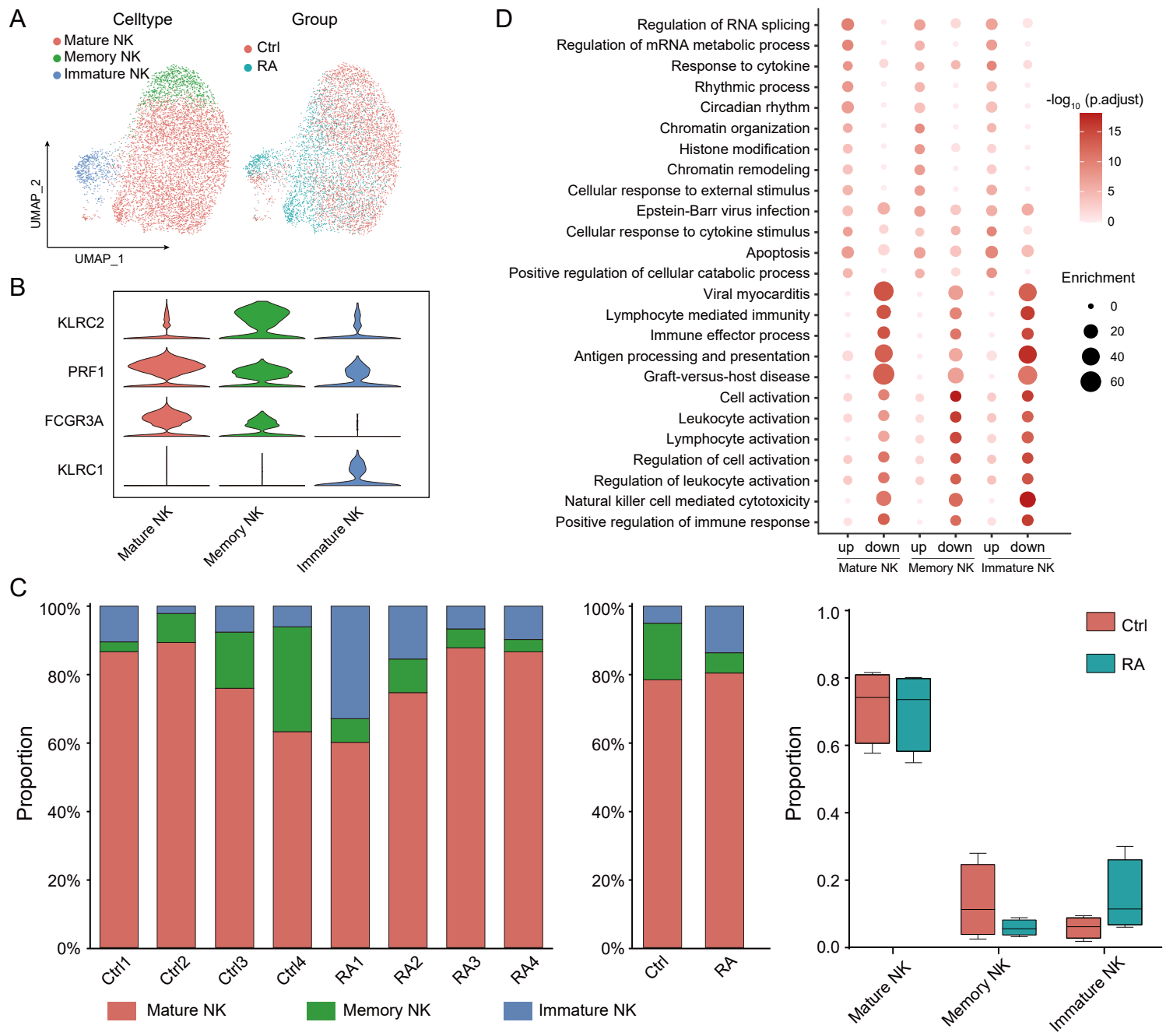

Supplement: Supplementary 14 — The scRNA profiles for NK cells in Ctrl and RA samples. [file 6300633.f14.pdf]

A

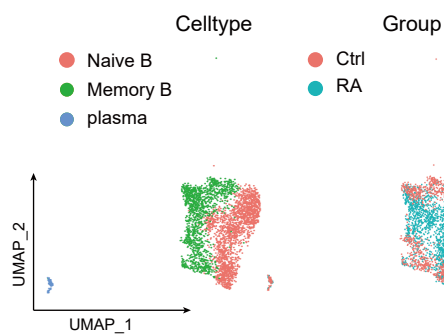

B

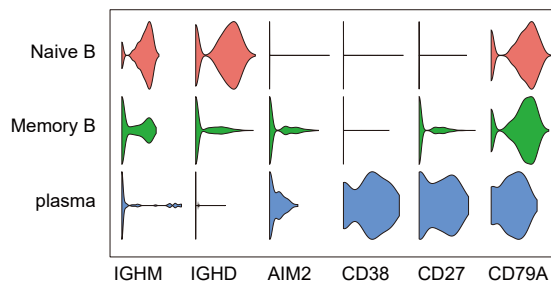

C

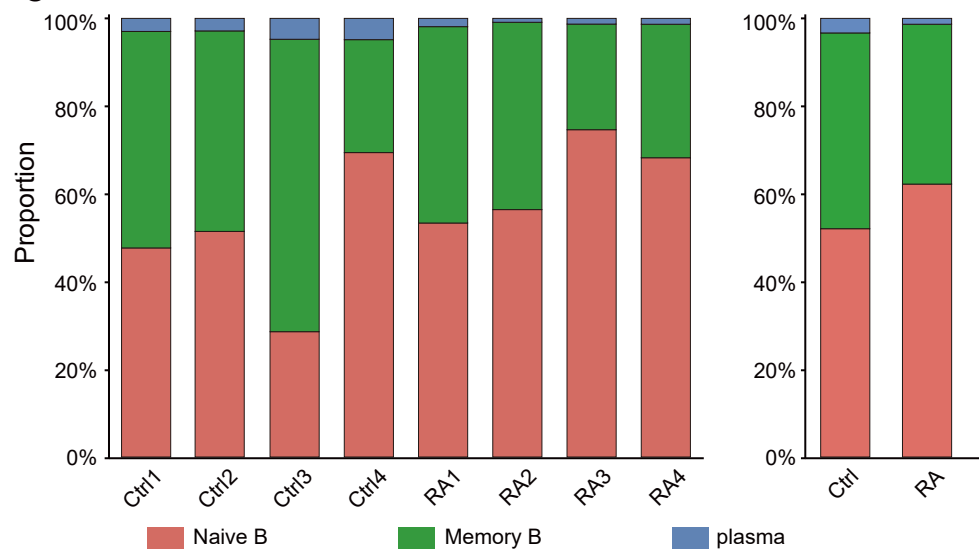

D

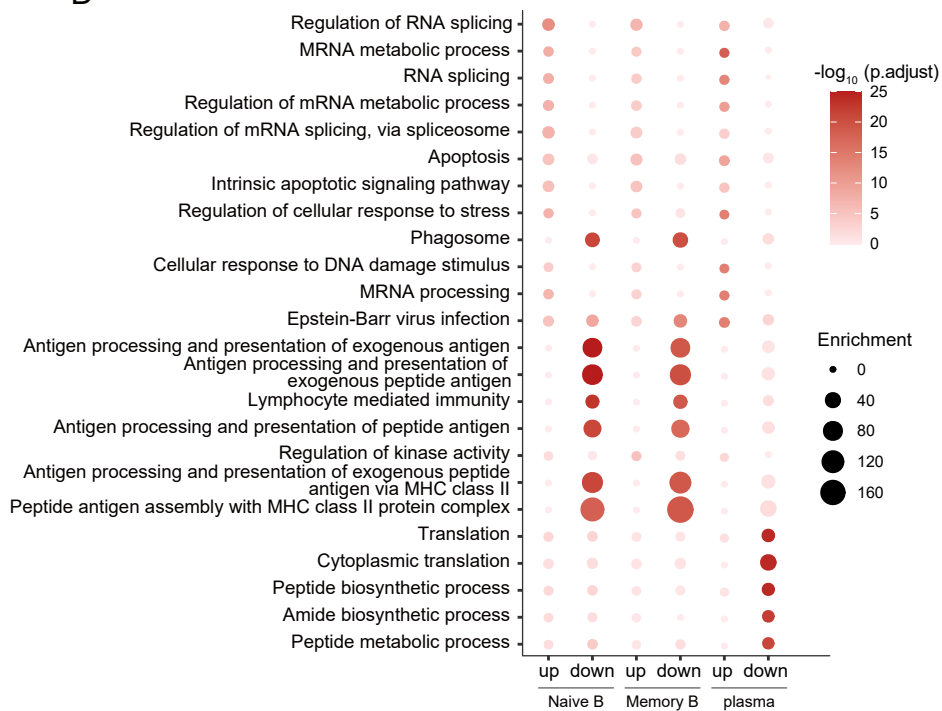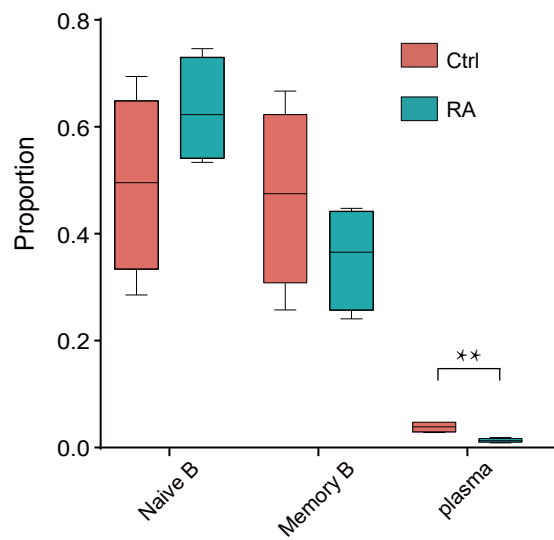

Supplement: Supplementary 15 — The scRNA profiles for B cells in Ctrl and RA samples. [file 6300633.f15.pdf]

**A**

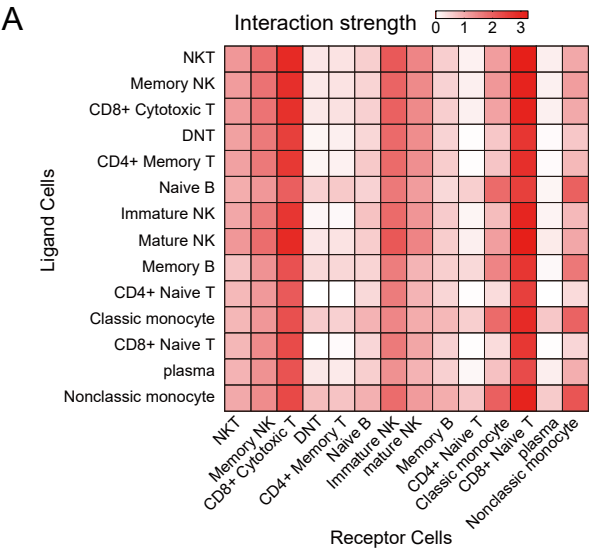

B

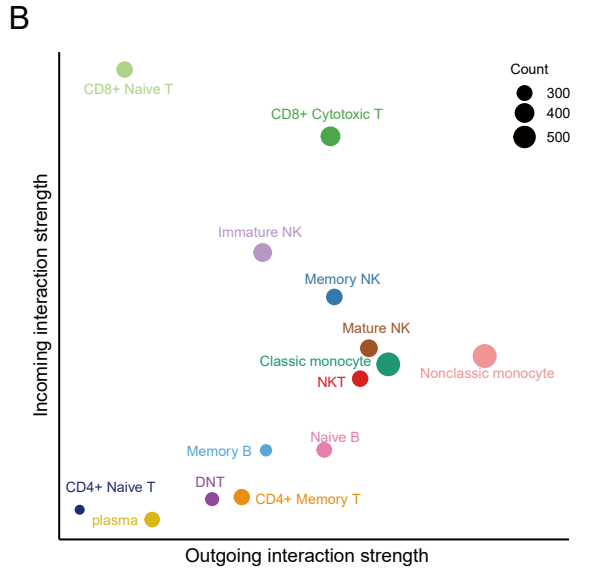

C

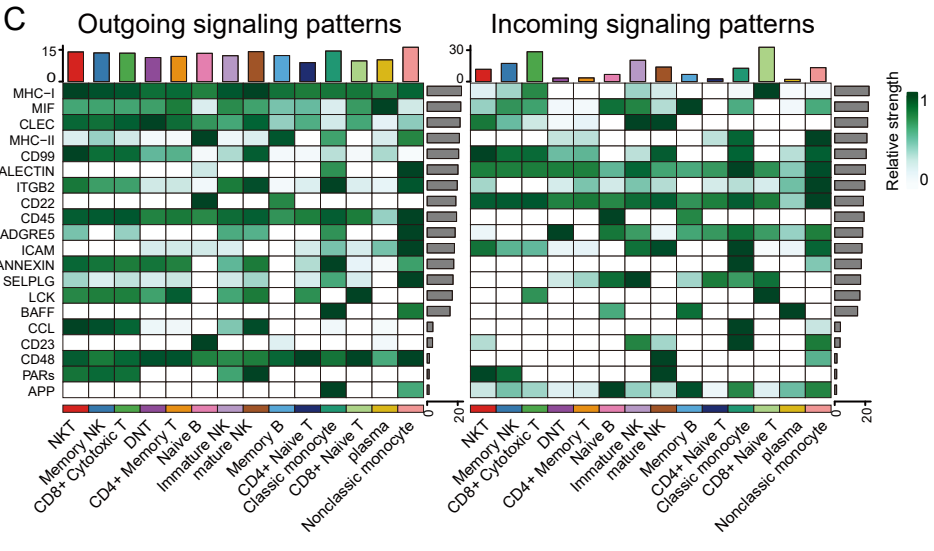

D

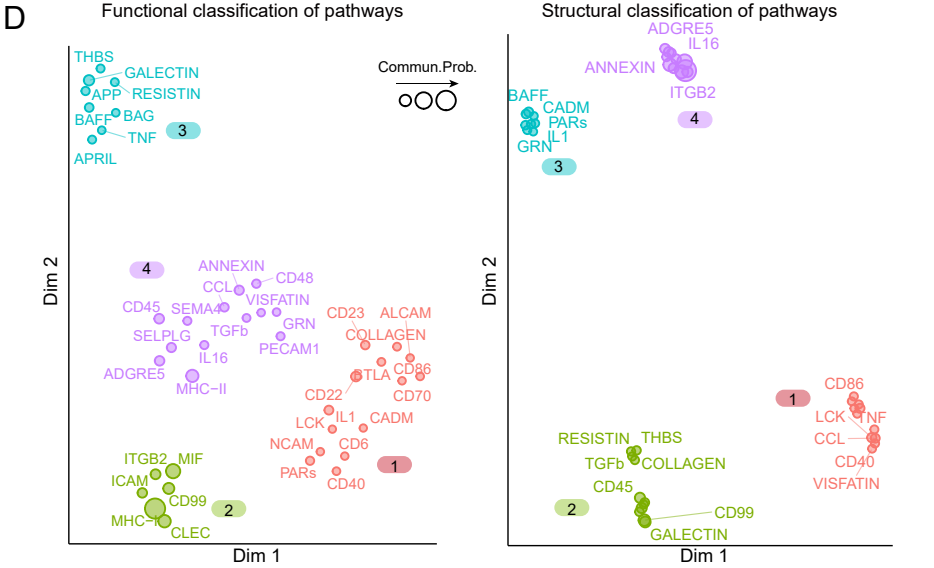

Supplement: Supplementary 16 — Communication networks between cell subpopulations in the PBMCs of Ctrl samples. [file 6300633.f16.pdf]

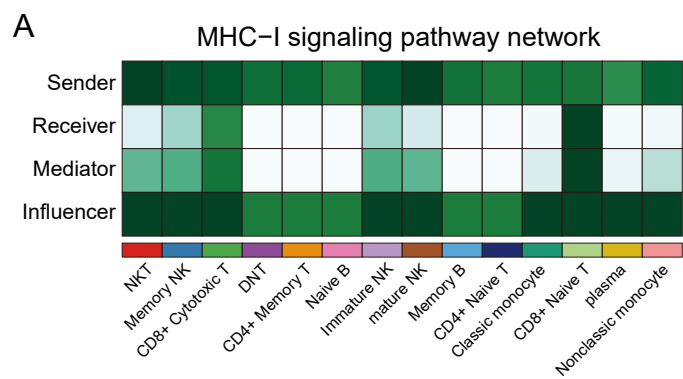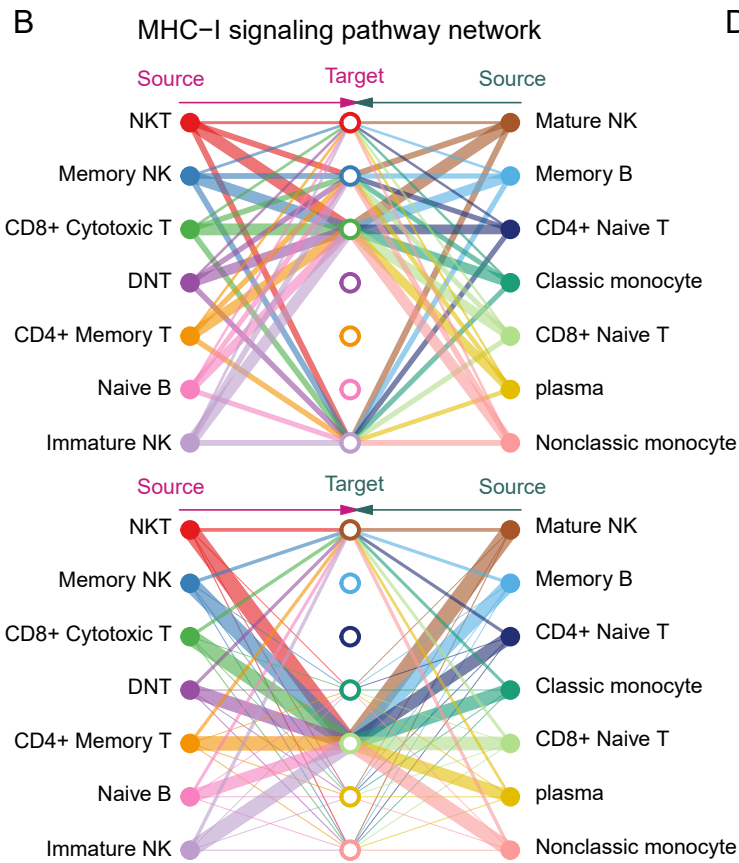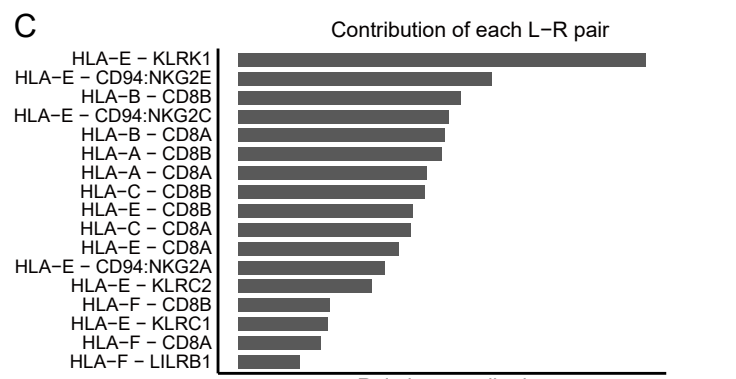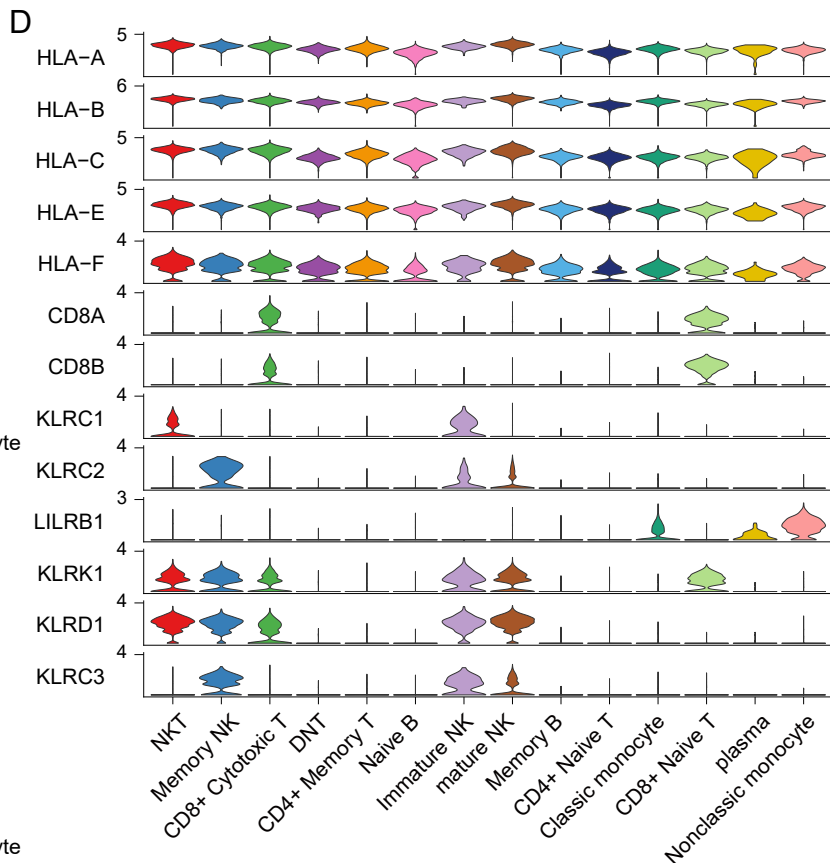

Supplement: Supplementary 17 — Network analysis of the MHC-I pathway in PBMCs from Ctrl samples. [file 6300633.f17.pdf]

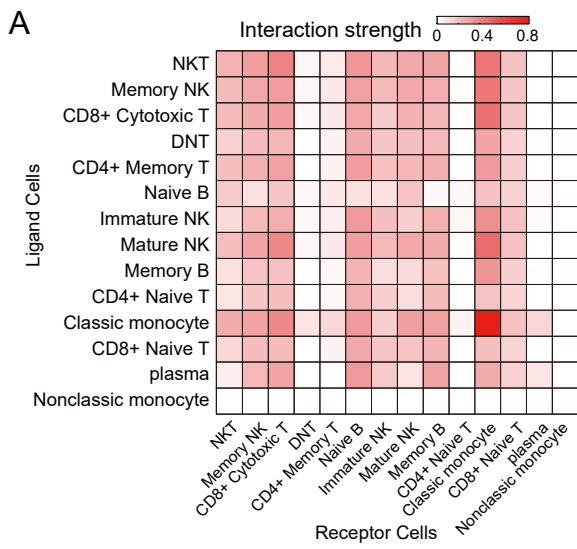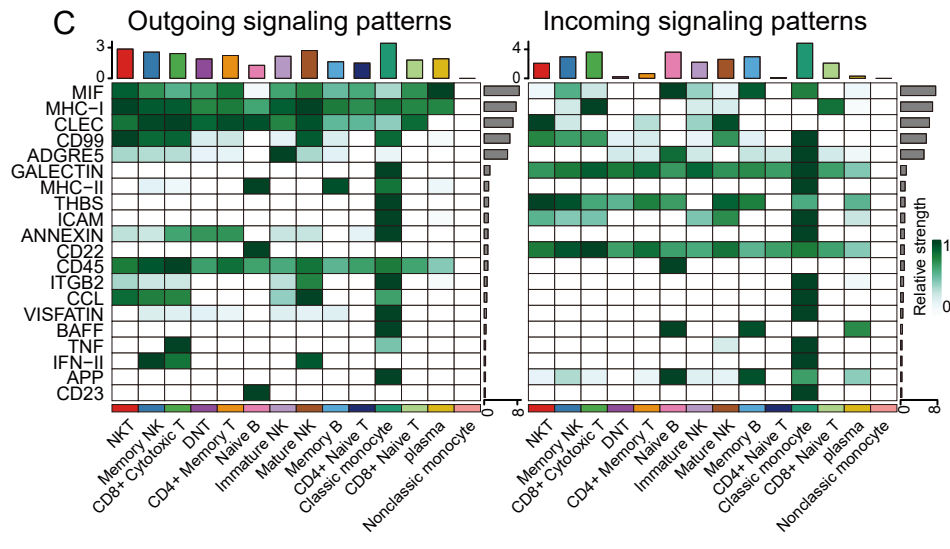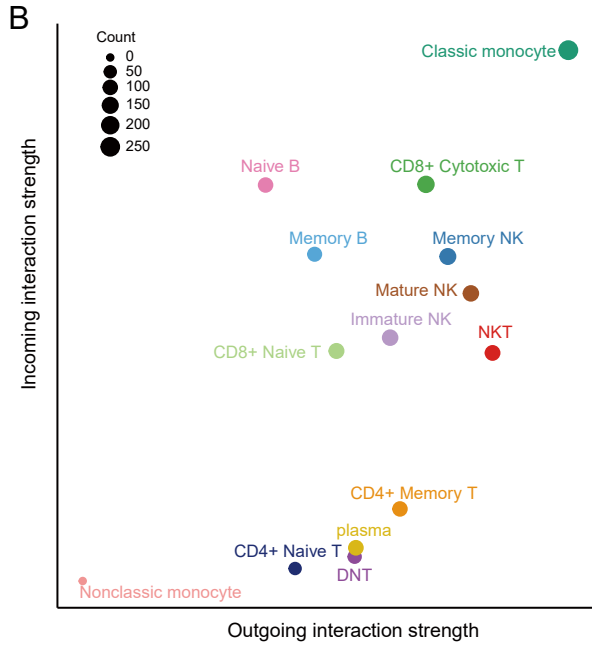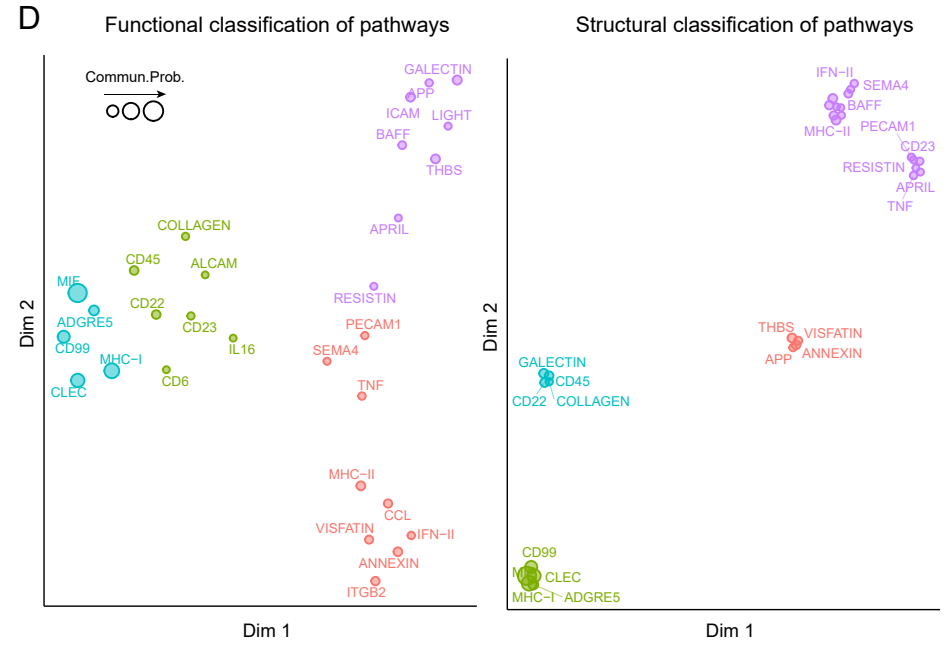

Supplement: Supplementary 18 — Communication networks between cell subpopulations in the PBMCs of RA patients. [file 6300633.f18.pdf]

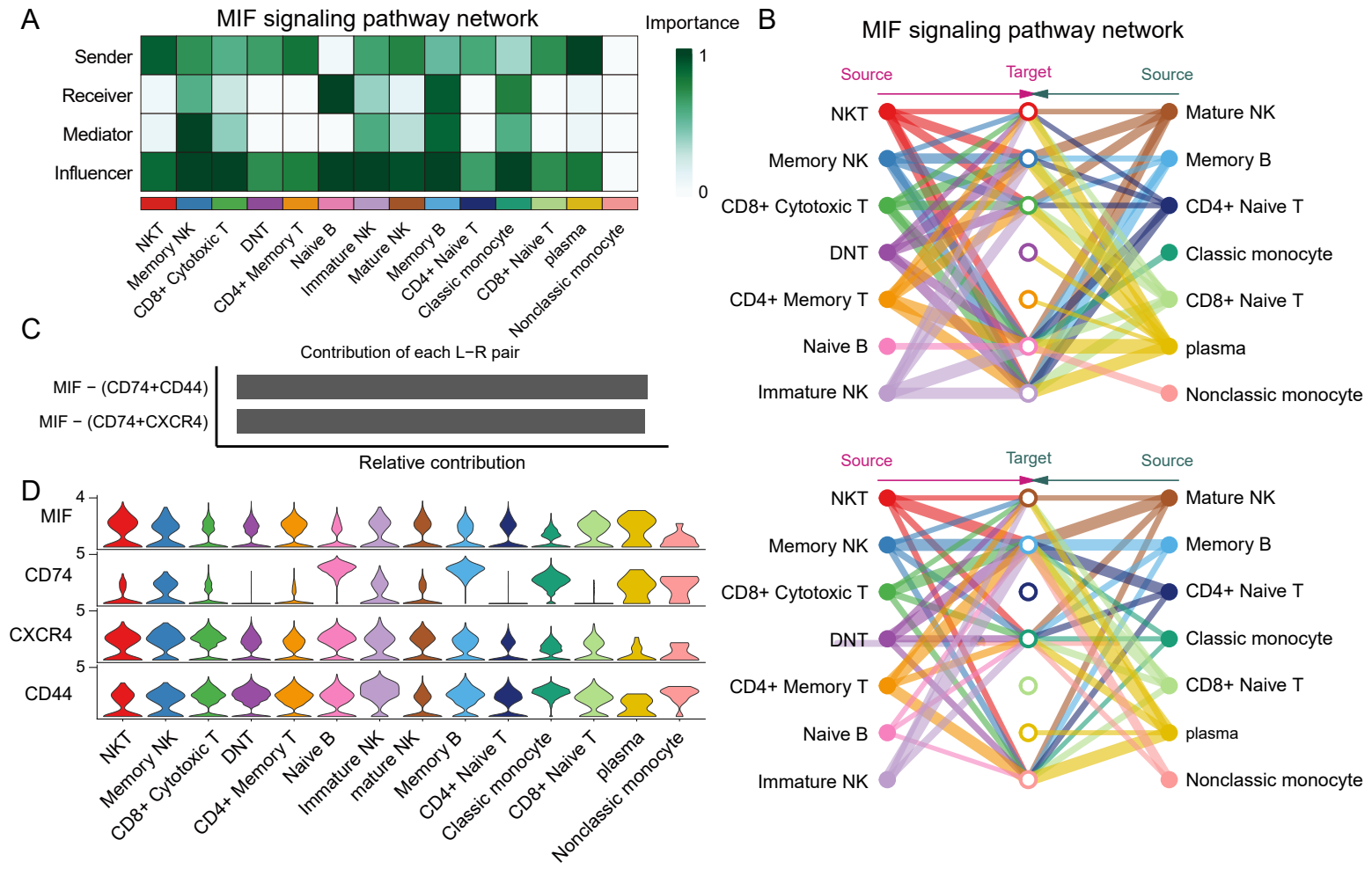

Supplement: Supplementary 19 — Network analysis of the MIF pathway in PBMCs from RA samples. [file 6300633.f19.pdf]

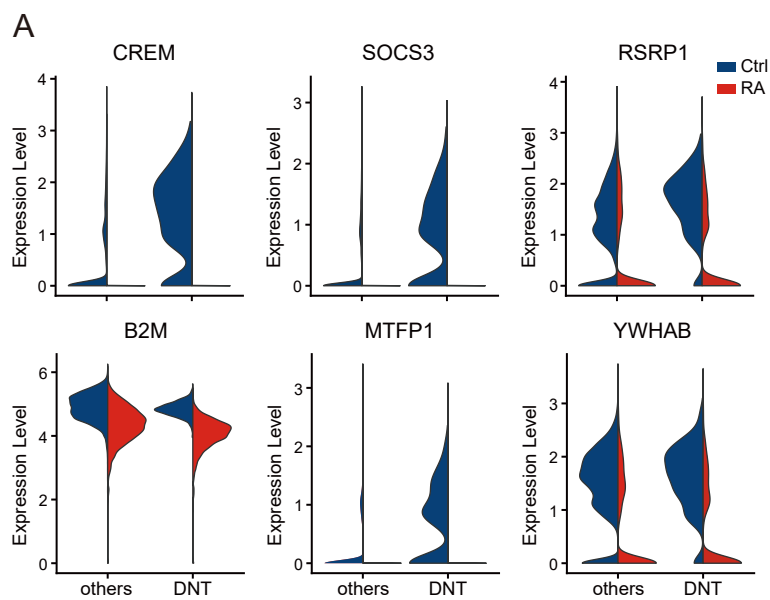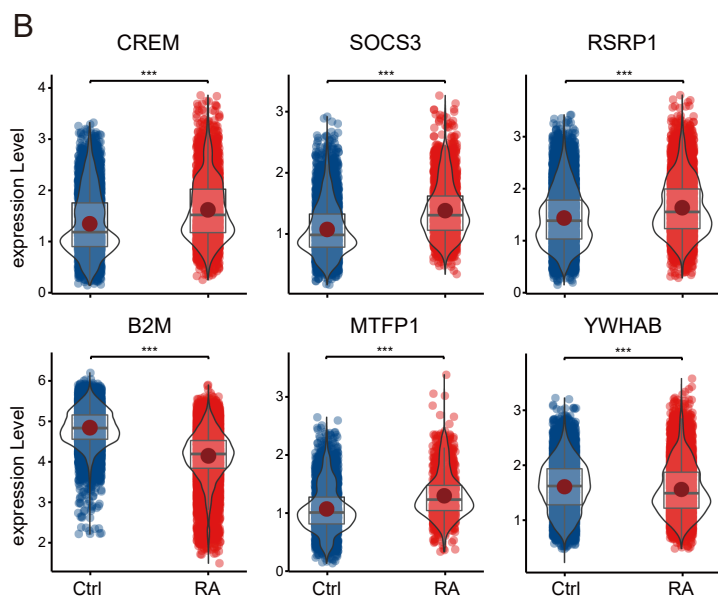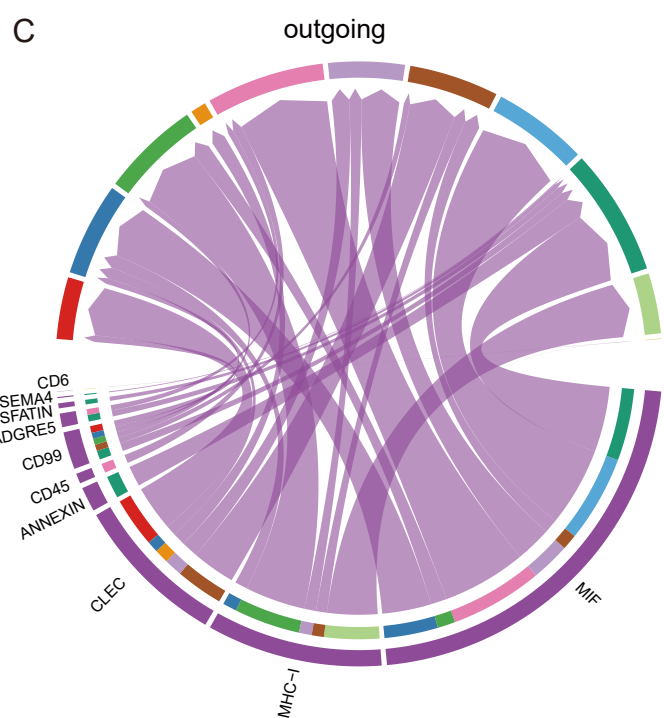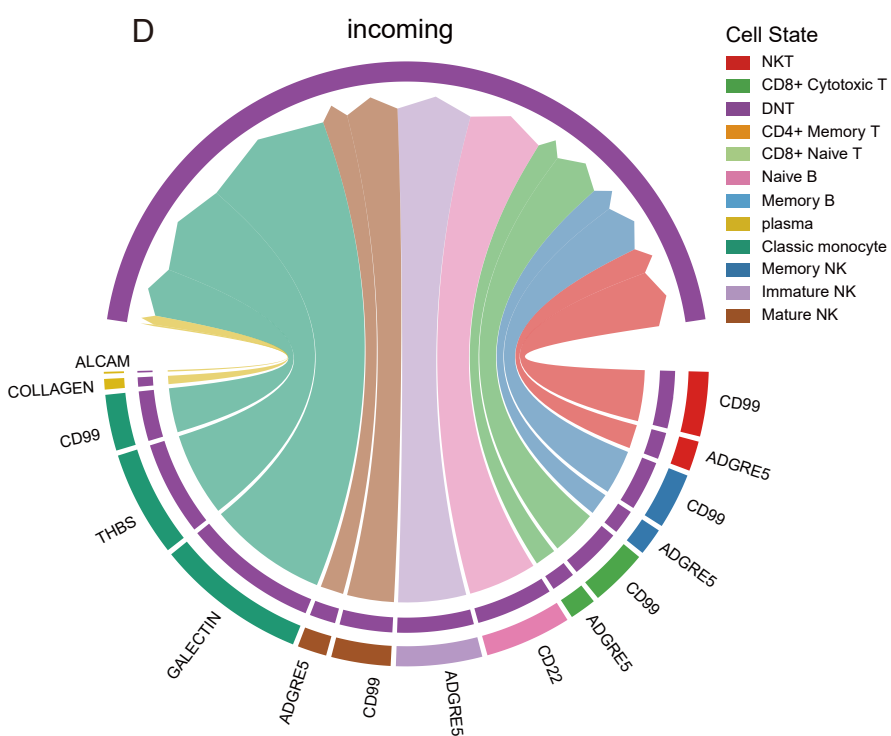

Supplement: Supplementary 20 — Analysis of DEGs and signaling pathways in DNT cells. [file 6300633.f20.pdf]

A

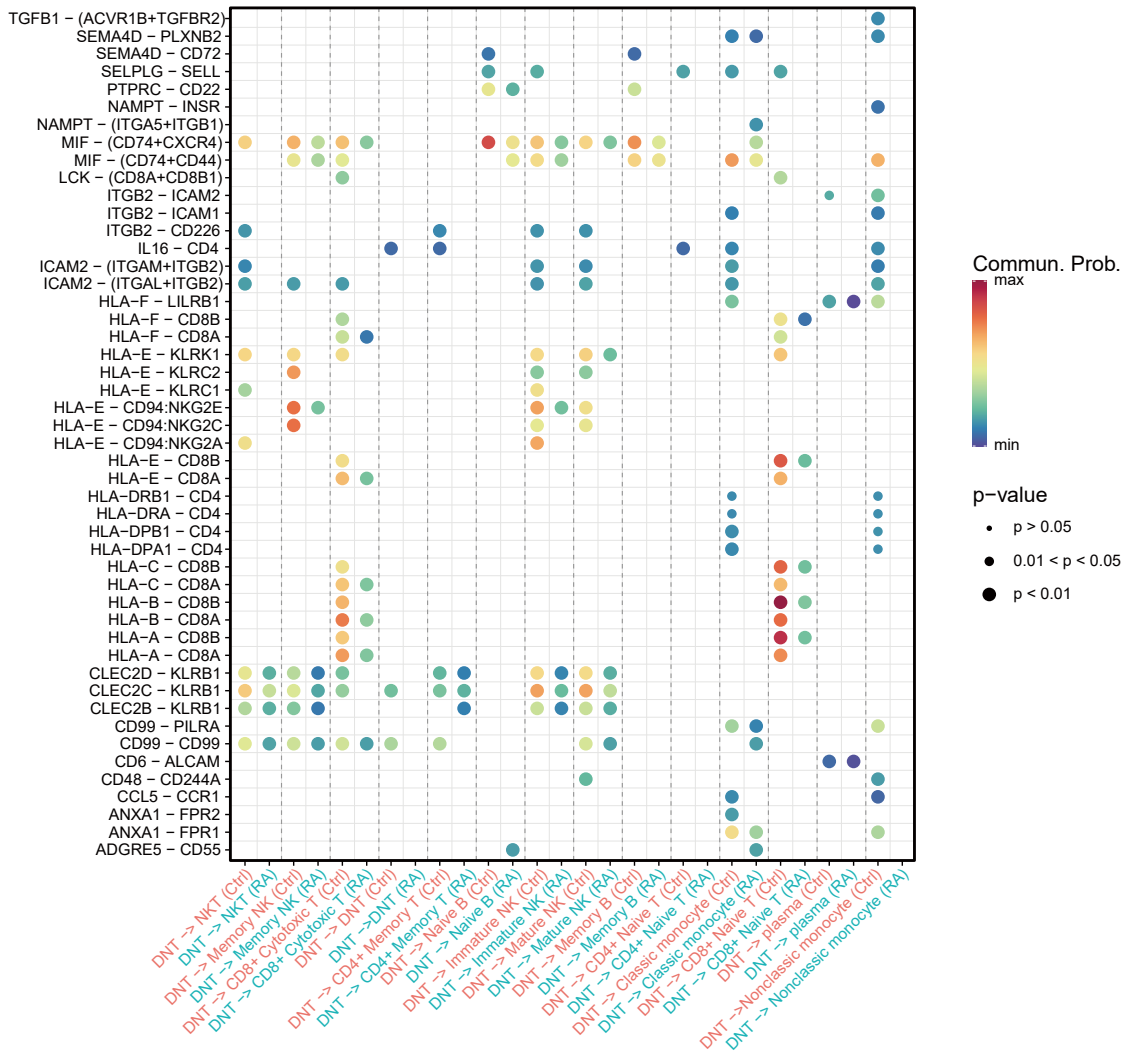

B

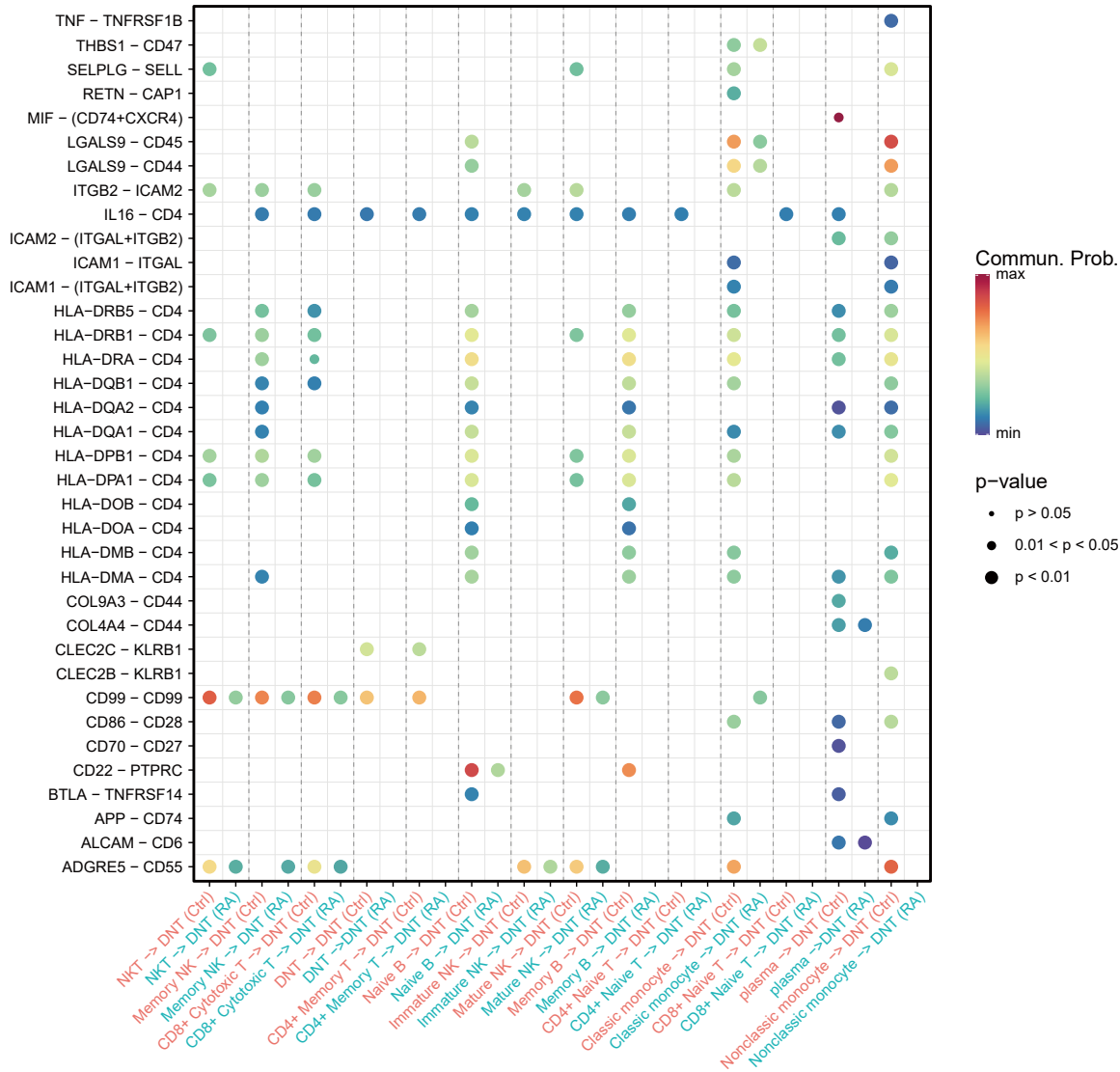

Supplement: Supplementary 21 — Dot plot showing the changing signaling ligand-receptor pairs between DNT cells and other cells. [file 6300633.f21.pdf]
